# 6^e^ journées des travaux scientifiques des soignant e s de Guyane. Nos soignant e s ont du talent ! 25 & 26 mai 2023, Cayenne, Guyane

**DOI:** 10.48327/mtsi.v3i4.2023.439

**Published:** 2023-10-31

**Authors:** Camille DESCHAMPS, Teddy BARDON, Théo BLAISE, Timothée BONIFAY, Mathilde BOUTROU, Alexis FREMERY, Kim HENRY, Yann LAMBERT, Paul LE TURNIER, Rémi MUTRICY, Margot OBERLIS, Benoît QUINTIN, Bénédicte SAUVAGE, Estelle THOMAS, Loïc EPELBOIN, Louise HUREAU-MUTRICY

**Affiliations:** 1 Service d'accueil des urgences, SAMU/SMUR, Centre hospitalier de Cayenne, Guyane, France; 2 Centre d'investigation clinique Antilles-Guyane, CIC INSERM 1424, Centre hospitalier de Cayenne, Guyane; 3 Unité sanitaire en milieu pénitentiaire de Rémire, Centre hospitalier de Cayenne, Guyane; 4 Unité des maladies infectieuses et tropicales, Centre hospitalier de Cayenne, Guyane; 5 Croix-Rouge française de Guyane; 6 Centre délocalisé de prévention et de soins de Saint-Georges-de-l'Oyapock, Centre hospitalier de Cayenne, Guyane; 7 BCom, Cayenne, Guyane; 8 COREVIH Guyane, Centre hospitalier de Cayenne, Guyane

**Keywords:** Soignants, Personnels de santé, Santé publique, Médecine tropicale, Épidémiologie, Santé sexuelle, Périnatalité, Zoonoses, Interculturalité, Guyane, Amérique latine, Caregivers, Health workers, Public health, Tropical medicine, Epidemiology, Sexual health, Perinatal care, Zoonoses, Interculturality, French Guiana, Latin America

**Figure 1:**
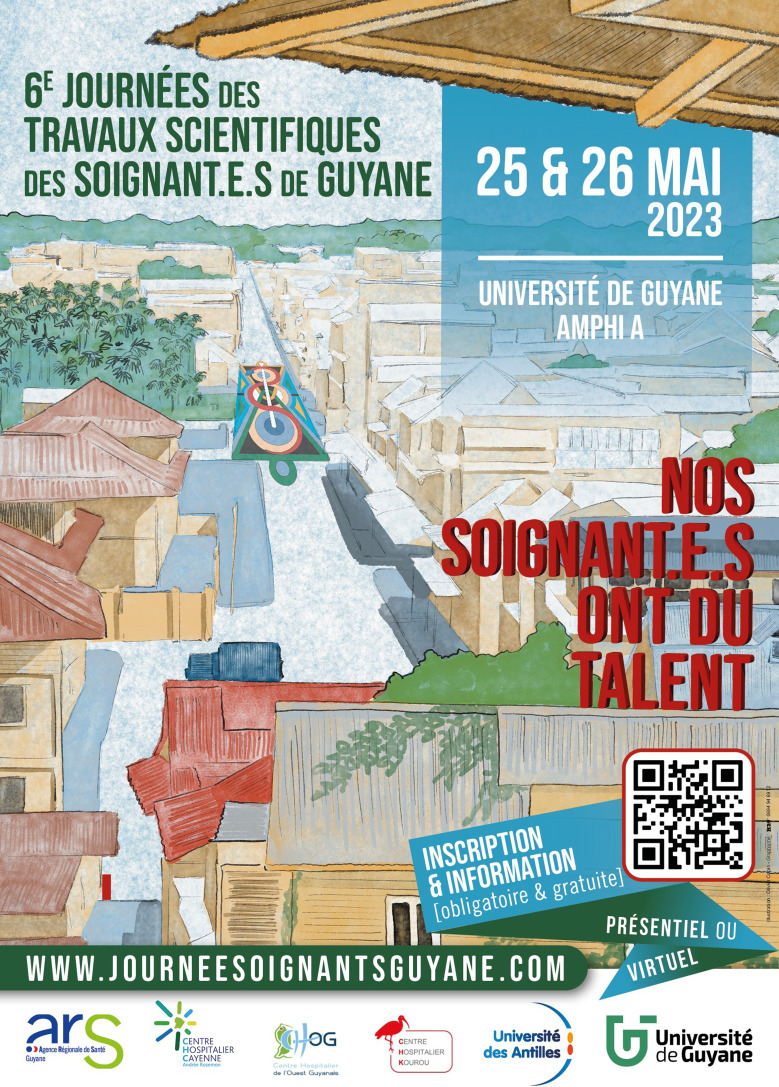
6^e^ journées des travaux scientifiques des soignant e s de Guyane. Nos soignantes ont du talent ! 25 & 26 mai 2023, Cayenne, Guyane Poster of the 6th day dedicated to the scientific works of caregivers in French Guiana. Our caregivers have talent! May 25 & 26, 2023, Cayenne, French Guiana

**Figure 2:**
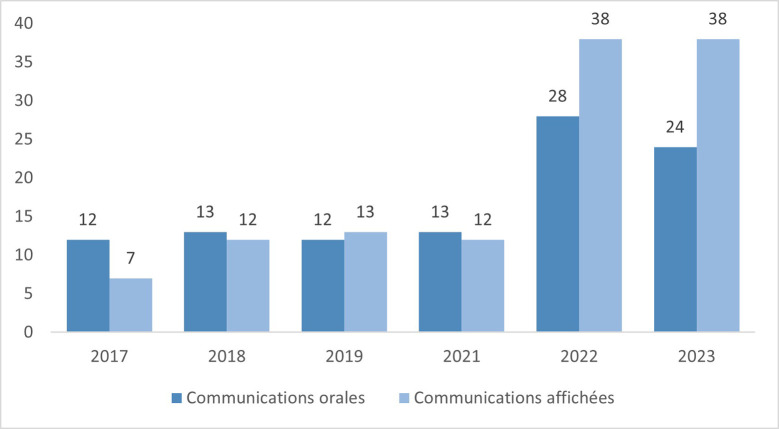
Eacute;volution du nombre de communications orales et affichées de 2017 à 2023 Number of oral and poster presentations from 2017 to 202

**Figure 3:**
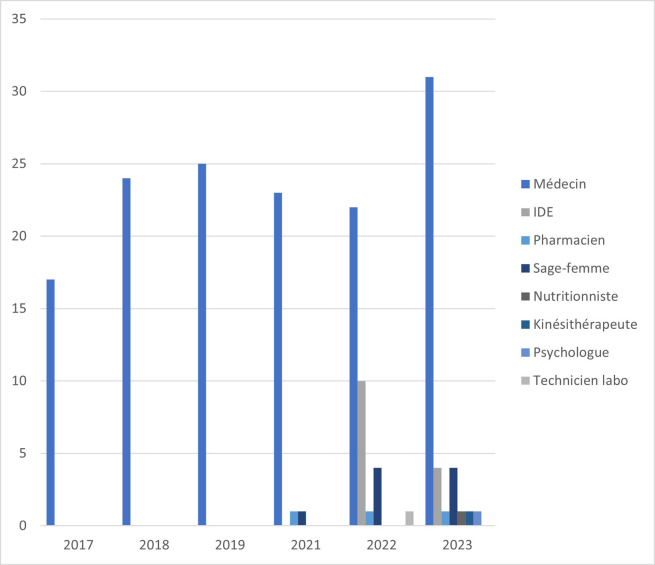
L’élargissement aux professionnelles de la santé non médicaux de 2017 à 2023 Extension to non-medical healthcare professionals from 2017 to 2023

L'université de Guyane a accueilli ces 25 et 26 mai 2023 les désormais incontournables « Journées des Soignant-e-s de Guyane » (Fig. 1). Cette 6e édition a mis en lumière des travaux sur des thématiques variées avec 24 communications orales et 38 communications affichées (Fig. 2). L’élargissement aux professionnel-le-s de la santé non médicaux, entamé depuis plusieurs années, s'est poursuivi avec, en plus des communications assurées par 28 médecins, celles des 3 infirmières, de la pharmacienne, des 4 sages-femmes, du nutritionniste, du kinésithérapeute et des 2 psychologues (Fig. 3).

L'infectiologie est toujours un sujet de prédilection, à travers des communications sur les zoonoses (toxoplasmose, leptospirose, maladie de Chagas), les pathologies vectorielles (paludisme et déficit en G6PD, syndrome alphagal, syndrome de Guillain-Barré), mais également les infections opportunistes liées au VIH avec au premier plan l'histoplasmose, ou encore les infections sexuellement transmissibles (VHB, antirétroviraux, IST et milieu carcéral). De nombreux sujets différents ont également été abordés touchant au genre et aux vulnérabilités (accompagnement psychologique, représentation des femmes trans, recours aux soins des travailleuses du sexe), à la précarité (kinésithérapie et Permanence d'accès aux soins de santé, phénomène des IVG itératives, précarité alimentaire des étudiants), au recours aux soins (soins antirabiques, motif de recours des CDPS aux urgences, cardiopathies aiguës aux urgences) ou à l'interculturalité et à la médiation (campagne binationale de dépistage, médecine traditionnelle, rôle de la médiation en santé).

**Figure 4:**
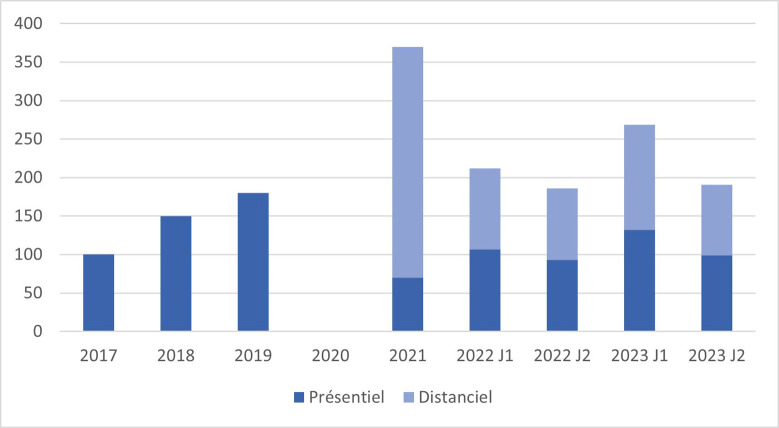
Répartition des participantes en distancie! et en présentiel de 2017 à 2023 Breakdown of remote and face-to-face participants from 2017 to 202

**Figure 5:**
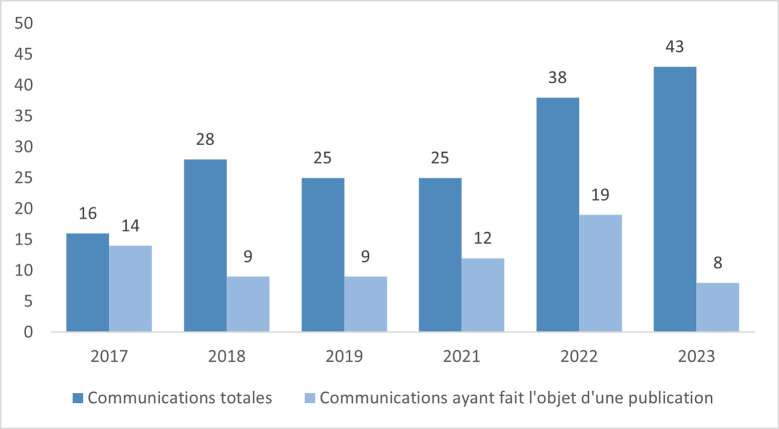
Communications aboutissant à des publications par des journaux scientifiques indexés de 2017 à 2023 Communications leading to publications in indexed scientific journals 2017 to 202

Le format mixte présentiel et/ou distanciel s'est révélé encore une fois un vrai succès: plus de 90 participant-e-s en distanciel et plus de 130 en présentiel (Fig. 4). Comme souligné au fil des années, les travaux présentés lors de ce congrès dépassent les frontières de la Guyane. Et chaque année, on note une augmentation des communications aboutissant à des publications au sein de journaux scientifiques internationaux. Cette année, 8 des 43 communications présentées ont déjà paru dans des journaux internationaux indexés (Fig. 5) !

**Figure 6:**
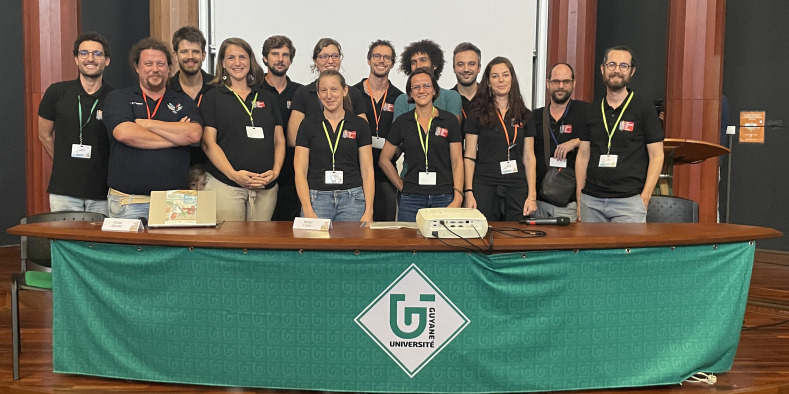
Le comité d'organisation du congrès, composé d'infirmier.e.s, d'une sage-femme, d'un pharmacien, d'une épidémiologiste et de médecins de diverses spécialités The conference organising committee, made up of nurses, a midwife, a pharmacist, an epidemiologist and doctors from various specialities

Note: Le contenu des résumés est l'expression du travail et de l'opinion de leurs auteur-e-s et ne reflète pas l'opinion du comité organisateur et scientifique du congrès.

Les grands témoins étaient cette année des personnels issus de l'université de Guyane, mais aussi du prestigieux GIRCI SOHO (Groupement interrégional de recherche clinique et d'innovation Sud-Ouest Outre-mer hospitalier) venus apporter leur soutien à ce manifeste de la recherche locale quelques mois après l'incorporation des hôpitaux de Guyane dans ce groupement. Ceux-ci ont souligné la qualité et l'originalité des travaux réalisés par la jeune génération de soignant-e-s de Guyane (Fig. 6). À nouveau, l'enthousiasme soulevé par la valorisation des travaux des soignant-e-s incite à poursuivre ces journées pour inspirer tous les acteurs et actrices de la santé en Guyane, notamment les plus jeunes.

