# Supplementary material for: 6th day dedicated to the scientific works of caregivers in French Guiana. Our caregivers have talent! May 25 & 26, 2023, Cayenne, French Guiana
Source: Med Trop Sante Int. 2023 Oct 31;3(4):mtsi.v3i4.2023.439. [Article in French] doi: 10.48327/mtsi.v3i4.2023.439 (PMC10879897; doi:10.48327/mtsi.v3i4.2023.439)
Supplement: Related texts

# Research article: Brucellose sur le plateau des Guyanes: émergence d'une nouvelle espèce?

**Published:** 2023-10-31

**Authors:** Frédégonde ABOUT, Théo PASTRE, Mathilde BOUTROU, Alex YAHIAOUI MARTINEZ, Alessia MELZANI, Sandrine PEUGNY, Céline MICHAUD, Sami ZOUAOUI, Thierry CARAGE, Vincent SAINTE-ROSE, Magalie DEMAR, Jean-Philippe LAVIGNE, Félix DJOSSOU, David O'CALLAGHAN, Loïc EPELBOIN, Anne KERIEL

**Affiliations:** 1 Centre hospitalier de Cayenne Andrée Rosemon, Cayenne, Guyane; 2 Centre hospitalier de Kourou, Kourou, Guyane; 3 Hôpital universitaire Carémeau, Nîmes; 4 Centre hospitalier de l'Ouest guyanais, Saint-Laurentdu-Maroni, Guyane; 5 Université de Guyane, Cayenne; 6 Université de Montpellier, Nîmes, Gard; 7 Institut national de la santé et de la recherche médicale (INSERM), Nîmes; 8 INSERM, Cayenne

## Introduction

La brucellose est une zoonose due aux bactéries du genre *Brucella,* dont les principales espèces virulentes chez l'homme sont *B. abortus, B. melitensis* et *B. suis.* Nous rapportons les trois premiers cas de brucellose sur le plateau des Guyanes et la découverte d'une nouvelle espèce de *Brucella.*

## Patients et méthode

La population d’étude portait sur les patients pris en charge dans l'un des 3 centres hospitaliers de Guyane, et reposait sur l'examen bactériologique. Les souches étaient ensuite envoyées au CNR des *Brucella* pour caractérisation par génétique moléculaire.

## Résultats

Trois cas ont été identifiés en 2017, 2020, et 2021 respectivement. Il s'agissait pour tous d'orpailleurs clandestins brésiliens récemment arrivés en Guyane après être passés par le Suriname voisin et vivant en forêt profonde. Ils étaient âgés respectivement de 29, 39 et 45 ans. Les tableaux cliniques étaient les suivants: fièvre nue avec céphalées et douleurs abdominales pour le premier (révélant également une infection par le VIH), douleurs lombaires fébriles pour le second, et un tableau d'altération de l’état général et de lombalgies fébriles évoluant depuis 10 mois, révélant une spondylodiscite L4-L5 avec épidurite et extension de l'abcès au psoas pour le troisième. Le diagnostic de brucellose a été fait par hémoculture pour les 2 premiers et par ponction de l'abcès du psoas pour le troisième. Les trois ont évolué favorablement sous rifampicine + doxycycline le temps de leur suivi.

L'analyse moléculaire des 3 isolats bactériens au CNR *Brucella* a confirmé le premier patient comme étant infecté par *B. suis* biovar 1. Pour les deux autres cas, les isolats sont génétiquement très proches et ne correspondent à aucune espèce de *Brucella* connue jusque-là. L'enquête auprès des 3 cas révèle comme facteur d'exposition potentiel la consommation de gibier chassé en forêt amazonienne. En outre, le premier élevait des porcs au Brésil, dans l’état du Maranhâo, ce qui est cohérent avec l'espèce *B. suis* isolée chez ce patient.

## Conclusion

Nous rapportons les 3 premiers cas de brucellose sur le plateau des Guyanes et la découverte d'une nouvelle espèce de *Brucella* probablement contractée au contact de la faune sauvage amazonienne et pour laquelle le nom de *Brucella amazoniensis* est proposé.

# Research article: Outils numériques et autisme, quel impact en Guyane?

**Published:** 2023-10-31

**Authors:** Amani ALEM

**Affiliations:** Pôle Santé mentale, Centre hospitalier de Cayenne

## Introduction

Les outils numériques offrent de nouvelles possibilités aux professionnels pour diversifier les pratiques. L'intérêt de ces technologies comme outil thérapeutique et éducatif est de plus en plus important de même que pour les chercheurs qui y consacrent de plus en plus d’études. Objectifs. À travers cette étude, il s'agit de proposer un état des lieux et une réflexion sur l'intégration des outils numériques dans un contexte spécifique en Guyane et d'acquérir une meilleure connaissance des usages des professionnels.

## Méthodes

Un questionnaire a été diffusé aux professionnels du service de pédopsychiatrie du Centre hospitalier Andrée Rosemon de Cayenne. Ce questionnaire est destiné aux professionnels accompagnant les enfants ou adolescents avec autisme dans l'usage des nouvelles technologies pour développer des compétences en autonomie, en communication et en interactions sociales.

## Résultats

Les résultats montrent que les professionnels ont recours à l'outil numérique pour permettre à l'enfant de développer certaines compétences. Les participants ont pu observer des effets importants auprès de certaines personnes avec trouble du spectre de l'autisme (TSA), principalement le gain en autonomie (26%), la formulation de demandes non verbales (21%) et l'accroissement du vocabulaire. 21% des soignants signalent que l'outil peut aider à exprimer ses émotions et à reconnaître celles des autres, 21% le qualifient comme une occasion pour les personnes avec autisme de répéter les mots et ainsi d'entrer en langage. La difficulté dans l'utilisation de l'outil numérique dans l'accompagnement des enfants avec TSA est due à plusieurs contraintes: 33,9% de notre échantillon mentionne les problèmes d'accès au réseau wifi et les restrictions techniques comme une contrainte majeure, 30% des enseignants touchés par notre enquête pensent qu'ils ne disposent pas de suffisamment de formation pour l'utilisation des outils numériques.

## Conclusion

Les domaines énumérés montrent le potentiel de ces moyens numériques et l'importance de leur maîtrise par les professionnels. Les obstacles cités ne devraient pas amener les professionnels à se passer des outils numériques, mais ils doivent être pris en compte pour un usage ancré dans la pratique.

# Research article: « Pourquoi tu vas voir la psy? » L'accompagnement psychologique à Saint-Georges: retour d'expérience clinique après deux années de pratique

**Published:** 2023-10-31

**Authors:** Anaïg AMOURET, Margaux BARBIER, Gilcelia ROUSSE, Lurdnalva LOPES, Léa BOURGUIGNON, Clémentine ÉTIENNE, Nadine GUILLAUME, Sophie RHODES

**Affiliations:** 1 Psychologue libérale, Caz'ID, IDsanté, Saint-Georgesde-l'Oyapock, Guyane; 2 Association IDsanté, Guyane

## Introduction

Dans le cadre du projet Oyapock Coopération Santé, une offre de consultations gratuites avec une psychologue clinicienne a été lancée en mars 2021 au sein de l'espace ressource Caz'ID (porté par l'association IDsanté) à Saint-Georges-de-l'Oyapock (SGO) grâce au soutien de l'ARS. Après deux années de pratique dans cette commune isolée, les caractéristiques du recours aux consultations et l'adhésion des patients mettent en évidence les enjeux du dispositif.

## Méthodes

Ce retour d'expérience s'appuie sur une collecte continue de données quantitatives et qualitatives sur les consultations et la patientèle depuis mars 2021, complétée par l'analyse des données cliniques.

## Résultats

200 patients issus de toutes les communautés ont été reçus entre mars 2021 et décembre 2022. La majorité n'avait jamais consulté de psychologue auparavant. Les premières consultations révèlent une difficulté à parler de soi, notamment en situation duelle et interculturelle. La médiation (culturelle et animale) joue alors un rôle important pour permettre une libération de la parole. 47% des demandes de suivi sont directement liées aux violences. De plus, le travail auprès des patients consultant pour dépression ou malêtre enfant/adolescent révèle que ces motifs sont souvent symptomatiques d'un traumatisme lié à des faits de violence. Quel que soit le profil des consultants, la violence est omniprésente. Elle se manifeste au niveau intrafamilial avec une forte représentation de personnes victimes de violences conjugales ou d'enfants exposés aux violences de leurs parents, au niveau extrafamilial avec des agressions multiples dans la commune, et enfin au niveau individuel avec une forte consommation de psychotropes et de nombreux passages à l'acte auto-agressifs.

## Discussion et conclusion

Ces consultations contribuent à repérer et verbaliser des situations de violences dans un cadre sécurisé et non stigmatisant. Elles permettent d'entamer un travail d’élaboration psychique destiné à endiguer le cycle de reproduction des schémas familiaux et sociétaux.

# Research article: Caractéristiques sociodémographiques, économiques et nutritionnelles des patientes atteintes de diabète gestationnel en Guyane: leviers ou freins dans l'observance des règles hygiéno-diététiques

**Published:** 2023-10-31

**Authors:** Lynda ANTOINE

**Affiliations:** Diététicienne-nutritionniste en Hôpital de jour de diabétologie, Centre hospitalier de Kourou, Guyane

## Introduction

Témoignant d'une prévalence importante, le diabète gestationnel (DG) des femmes guyanaises est estimé à 10,3%. Le territoire est caractérisé par sa diversité culturelle et la précarité de sa population, ce qui a un impact sur la bonne observance des règles hygiéno-diététiques.

## Matériel et méthode

Une étude qualitative a été menée avec un recueil de données lors d'entretiens qui se sont déroulés à l'hôpital de Kourou de février à mai 2022, avec 15 patientes âgées de 23 à 40 ans d'origines ethniques et socio-professionnelles diverses. L'objectif est d'analyser les facteurs déterminants d'une bonne observance ou non des règles hygiéno-diététiques.

## Résultats

77% de la population étudiée estiment un changement de régime alimentaire plus sain trop onéreux. Les habitudes alimentaires indiquent que 86% des patientes ont une perception erronée d'une alimentation équilibrée et du rythme alimentaire. 60% des patientes n'ont pas ou peu de connaissances de la pathologie. 67% considèrent la surveillance glycémique et l’équilibre alimentaire comme trop contraignants, et 67% ne pratiquent pas d'activité physique.

## Conclusions

L'importance du respect de l'observance des règles hygiéno-diététiques n'a pas recueilli l'unanimité. La précarité et le manque de connaissances de la maladie en sont les facteurs essentiels.

# Research article: La maladie de Chagas: où en sommes-nous? Étude épidémiologique des cas diagnostiqués en Guyane entre 2008 et 2018

**Published:** 2023-10-31

**Authors:** Sophie ARNAUDIN, Philippe ABOUD, Magalie DEMAR, Félix DJOSSOU, Loïc EPELBOIN, Mathieu NACHER, Céline MICHAUD, Denis BLANCHET, Mélanie GAILLET

**Affiliations:** 1 Médecin généraliste, La Réunion, France; 2 Service des maladies infectieuses et tropicales, Centre hospitalier de Cayenne, Guyane; 3 Laboratoire, Centre hospitalier de Cayenne; 4 Tropical Biome & Immunophysiopathology (TBIP), Université de Guyane, Guyane; 5 Centre d'investigation clinique Antilles-Guyane, CIC INSERM 1424, Guyane; 6 Centres délocalisés de prévention et de soins (CDPS), Guyane; 7 Laboratoire d'analyse médicale, Cayenne; 8 Service d’épidémiologie et d’évaluation médicale, Centre hospitalo-universitaire de Grenoble-Alpes, Grenoble, Isère

## Introduction

Les difficultés de prise en charge de la maladie de Chagas représentent un obstacle aux soins des patients et posent des problèmes de santé publique. Méthode. Il s'agissait d'une étude épidémiologique, rétrospective, multicentrique. Les caractéristiques sociodémographiques, cliniques, paracliniques des patients ayant eu un test biologique Chagas positif entre 2008 et 2018 en Guyane ont été collectées. Les patients ont été comparés selon que les cliniciens avaient retenu ou non le diagnostic de maladie de Chagas.

## Résultats

Parmi les 247 patients inclus, 38 (15,4%) ont eu un diagnostic de maladie de Chagas retenu par les cliniciens. L’âge moyen était de 48,1 ans [ET = 17,0] vs 39,5 ans pour les non-retenus (NR) [ET = 18,8] (p = 0,01). Le sex-ratio était de 2,17 *vs* 1,43 pour les NR, 44.7% étaient nés au Brésil (n = 17/38) *vs* 18.7% (n = 39/209) chez les NR (p < 0,01). Parmi les patients retenus, 18,4% (n = 7/38) vivaient à l’étranger, *vs* 1,9% des NR (n = 4/209) (p < 0,01). Parmi les cas retenus, on comptait 8 formes aiguës, 29 chroniques dont 6 indéterminées, 20 déterminées (18 formes cardiaques, 2 formes digestives), 3 réactivations. Parmi les formes chroniques, 14 patients présentaient une cardiomyopathie dilatée (48,3%). Un traitement antiparasitaire avait été administré à 55,2% des patients (n = 21/38). Parmi les 209 cas NR, un diagnostic différentiel avait été posé pour 66,5% des patients (n = 139/209).

## Discussion et conclusion

Le diagnostic de la maladie de Chagas reste complexe. Le renforcement des actions de prévention, la mise à jour des protocoles, le déploiement de nouveaux outils diagnostiques, la consolidation des circuits de soins permettront d'améliorer la lutte contre la maladie de Chagas et ses conséquences.

# Research article: G-VOC: Étude de la gravité du variant gamma du SARS-CoV-2 comparé aux variants antérieurs (virus historique, alpha et zêta) en Guyane

**Published:** 2023-10-31

**Authors:** Alain-Bruno BARDIGUYO, Nicolas VIGNIER, Mohamed LARBI, Dominique ROUSSET, Frédégonde ABOUT, Camille THOREY, Mathilde BOUTROU, Amadou Oury BALDÉ, Marc LEDY, Christelle ELFORT, Jean-François CAROD, Thierry CARAGE, Magalie DEMAR, Maëlle LE GOFF, Rachel ROBERT, Audrey PHIBEL, Mayka MARGEAY-FABRE, Charlotte CASADEPAX-SOULET, Maylis DOUINE, Mathieu NACHER, Félix DJOSSOU, Anne LAVERGNE, Théo BLAISE, Antoine ENFISSI, Loïc EPELBOIN

**Affiliations:** 1 Unité des maladies infectieuses et tropicales, Centre hospitalier de Cayenne, Guyane; 2 COREVIH Guyane, Centre hospitalier de Cayenne; 3 CIC INSERM 1424, Centre hospitalier de Cayenne; 4 CNR virus respiratoires, Institut Pasteur de la Guyane; 5 Service de médecine, Centre hospitalier de Kourou; 6 Service de médecine, Centre hospitalier de l'Ouest guyanais; 7 Laboratoire Biosoleil, Cayenne; 8 Laboratoire de microbiologie, Centre hospitalier de l'Ouest guyanais; 9 Laboratoire d'analyse médicale, Kourou; 10 Laboratoire hospitalo-universitaire de microbiologie, Centre hospitalier de Cayenne

## Introduction

La Guyane, du fait de sa situation géographique en Amérique du Sud et de ses liens permanents avec l'Hexagone, a été exposée à des vagues épidémiques de Covid-19 venant tantôt de l'Europe, tantôt du Brésil. Début 2021, le variant gamma arrivé du Brésil a très rapidement remplacé le virus historique, avec une morbi-mortalité apparente accrue. La gravité de ce variant a été peu étudiée en dehors du Brésil. L'objectif primaire de notre étude était de comparer la gravité clinique des Covid-19 dues au variant gamma par rapport aux autres variants circulant en Guyane sur la même période.

## Méthode et matériels

Une étude rétrospective multicentrique a été réalisée sur la période de janvier à avril 2021. Tous les patients infectés par SARS-CoV-2 ayant bénéficié d'un séquençage étaient incluables dans l’étude. Il s'agissait de patients pris en charge dans l'un des trois centres hospitaliers (Cayenne, Kourou, Saint-Laurent), dans les Centres délocalisés de prévention et de soins, et en ville. Les patients non joignables et les patients refusant de participer à l’étude n'ont pas été inclus. Les données sociodémographiques et cliniques ont été recueillies. Le critère de jugement principal était le décès et les critères secondaires comprenaient l'hospitalisation, le séjour en réanimation, et le besoin d'une oxygénothérapie. Les patients infectés par le variant gamma (20J/501Y.V3) ont été comparés aux autres variants regroupés: virus historique (19B, 20A, 20A.EU2 et 20E (EU1)), variant alpha (20I/501Y. V1) et variant zêta (20B/P2 – 484K). Les variables continues ont été comparées en utilisant le test de Student et les variables catégorielles en utilisant le test exact de Fischer. Résultats exprimés en pourcentage (seuil de significativité < 0,05; odds ratio brut et intervalle de confiance à 95%). Une hospitalisation était définie comme un séjour à l'hôpital ± 2 nuits.

## Résultats

Parmi les 380 patients éligibles, 339 patients ont été inclus dans l’étude et 41 non inclus (5 non joignables et 36 refus). L’âge moyen était de 39,2 ± 17,7 ans, 48,7% étaient des femmes, et 20,7% avaient au moins une comorbidité. Cinquante patients ont été hospitalisés (14,7%) dont 16 en réanimation (4,7%) et 9 (2,7%) sont décédés. On ne retrouvait pas de différence d’âge significative entre les 2 groupes, ni de fréquence de l'HTA, du diabète, de l'obésité ou d'autres comorbidités. Les patients infectés par le variant gamma ont été plus souvent hospitalisés que le groupe témoin (20,6% *vs* 10,4%; p = 0,01; OR = 2,2 (1,2-4,4)) et ont plus souvent eu besoin d'une oxygénothérapie (11,6% vs 2,6%, p = 0,01; OR = 4,9 (1,7-17,5). Il n'y avait pas de différence significative en termes d'oxygénation invasive (haut débit ou intubation) (4,8% vs 2,6%; p = 0,21), d'hospitalisation en réanimation (6,9% *vs* 3,1%; p = 0,12), ou de décès (3,4% *vs* 2,1%; p = 0,5).

## Conclusion

Le variant gamma ne semble pas avoir entraîné une plus forte mortalité liée à la Covid-19 en comparaison avec les autres variants circulant en Guyane à la même période dans le contexte particulier de la région (population jeune et faiblement préexposée au virus). Cependant, on a observé plus d'hospitalisation et d'oxygéno-requérance dans le groupe gamma. L'absence de recrutement des cas de Covid-19 non hospitalier est aussi à prendre en compte. D'autres études sur des effectifs plus importants sont nécessaires pour confirmer ou infirmer ces différences.

# Research article: Hyalinose segmentaire et focale au cours du syndrome Nail-Patella: à propos d'un cas

**Published:** 2023-10-31

**Authors:** Malika BELGRINE, Ronan HOUITTE, Mohamed SIDIBE, Samiratou TAPSOBA, Timote DAVODOUN, Tanguy-Fortuney GBAGUIDI

**Affiliations:** 1 Service de néphrologie-dialyse, Centre hospitalier de Cayenne; 2 Service de médecine interne, Centre hospitalier de Cayenne

## Introduction

Le syndrome Nail-Patella (NPS) est une maladie rare, autosomique dominante liée à des mutations du gène *LMX1B.* Il se caractérise par des ongles dysplasiques, des rotules absentes ou hypoplasiques, une dysplasie du coude et des cornes iliaques. L'atteinte rénale détermine le pronostic du NPS. Les patients présentent souvent des degrés divers de protéinurie ou d'hématurie, et peuvent parfois évoluer vers une insuffisance rénale chronique. Nous rapportons ici un cas de hyalinose segmentaire et focale cortico-résistante associée au NPS.

## Présentation du cas

Une femme de 29 ans, connue du service pour syndrome néphrotique depuis 2018, traitée par corticoïde 1 mg/kg/j, initialement non observante. Reprise des corticoïdes en 2019 devant la persistance du syndrome néphrotique, avec un suivi toujours aléatoire et perte de vue. En janvier 2021, la ponction biopsie rénale retrouvait une hyalinose segmentaire et focale péri-hilaire, avec dépôts de C3 et d'IgM, justifiant la reprise de la corticothérapie à 1 mg/kg, qui s’était compliquée d'un diabète insulino-requérant. En juin 2021, elle était réhospitalisée pour persistance du syndrome néphrotique. Elle avait une dysplasie des ongles, des coudes bloqués en flexion, un faciès cushingoïde. L'interrogatoire retrouvait un antécédent d'ongles dysplasiques chez son père, et des coudes bloqués chez son frère. Au bilan biologique, persistance du syndrome néphrotique et du diabète cor tico-induit, sans insuffisance rénale ni anomalies immunologiques. À l'imagerie il y avait luxation, hypoplasie des têtes radiales et hypoplasie des patellas. Devant ce tableau, suspicion de syndrome Nail-Patella, motivant l'arrêt de la corticothérapie, et étude génétique montrant une mutation hétérozygote du gène *LMX1B* en c.691c>T, situé au niveau de l'exon 4.

## Conclusion

L’étude génétique est indispensable devant toute hyalinose segmentaire et focale cortico-résistante, surtout en cas de présence de signes extra-rénaux évocateurs ou d'antécédents familiaux, afin d’éviter les complications de l'immunosuppression, comme c'est le cas de notre patiente qui avait développé un diabète cortico-induit et une insuffisance surrénalienne.

# Research article: Enquête de satisfaction auprès des usagers de la Permanence d'accès aux soins de santé et des Équipes mobiles d'accompagnement et de soins du Centre hospitalier de l'Ouest guyanais

**Published:** 2023-10-31

**Authors:** Emmanuel BEKOLO, Camille OBERT-MARBY

**Affiliations:** 1 Maison de santé pluridisciplinaire Léopold, Saint-Laurent-du-Maroni, Guyane; 2 Département universitaire de médecine générale, Université des Antilles, Pointe-à-Pitre, Guadeloupe; 3 Service de la PASS, Centre hospitalier de l'Ouest guyanais, Saint-Laurent-du-Maroni

## Introduction

Précarité, barrière culturelle et répartition inégale de l'offre de soins sur le territoire constituent les principales difficultés d'accès à l'offre de soins de la population de l'Ouest guyanais. La Permanence d'accès aux soins de santé (PASS) et les Équipes mobiles d'accompagnement et de soins (EMAS) sont deux services du Centre hospitalier de l'Ouest guyanais (CHOG) qui s'inscrivent dans une logique d’« aller vers » pour pallier ces problèmes. Ayant été déployés récemment, ces services font l'objet d'une évaluation des pratiques.

## Méthodes

Étude quantitative prospective descriptive couplée à une évaluation qualitative de la satisfaction par auto-questionnaire à questions ouvertes. L'objectif principal de cette étude est d’évaluer le degré de satisfaction des usagers. Les objectifs secondaires sont de déterminer les domaines d'insatisfaction et de dégager des axes d'amélioration. Les usagers inclus étaient majeurs, volontaires, maîtrisant l'une des sept langues disponibles du questionnaire, sans comorbidité psychiatrique ou démence.

## Résultats

200 questionnaires recueillis pour la PASS sur la période du 13 avril au 5 mai 2022 (11 vacations), 20 questionnaires pour les EMAS du 8 avril au 13 mai 2022 (4 vacations). Très forte satisfaction globale des usagers des deux services. Les principaux domaines d'insatisfaction concernent les informations sur le fonctionnement des deux services, l'orientation pour les problèmes sociaux non en lien avec l'accès aux droits de sécurité sociale, le temps d'attente en salle d'attente spécifique (PASS) et l'information et la mise en relation avec les professionnels libéraux spécifiques (EMAS).

## Discussion et conclusion

Ce travail a mis en exergue l'importance de l'ouverture de nouvelles lignes de consultation sur rendezvous pour la PASS, et de la transmission d'informations documentées sur les missions des deux services. Le retour aux droits communs des usagers des EMAS est à anticiper. Une future enquête qualitative pure serait souhaitable pour mieux recueillir les problématiques émanant directement des usagers.

# Research article: Qui sont les femmes trans en Guyane?

**Published:** 2023-10-31

**Authors:** Chloé BERTIN, Aude LUCARELLI, Félix DJOSSOU, Jade GHOSN, Marc DIGUMBER, Nicolas VIGNIER

**Affiliations:** 1 Centre hospitalier universitaire de Bichat-Claude Bernard, Paris; 2 Centre hospitalier de Cayenne, Guyane; 3 COREVIH Guyane; 4 COREVIH Île-de-France Nord

## Introduction

Il n'existe pas de travaux sur les femmes trans en Guyane, mais de nombreuses publications émanent de pays proches géographiquement (Brésil, Pérou, États-Unis), ou de métropole. Pourtant, les violences subies et les risques infectieux sont élevés chez ces femmes ayant fréquemment recours au travail du sexe (TDS). En effet, elles porteraient, proportionnellement, le fardeau le plus lourd en matière d'infection VIH dans le monde et notamment en Amérique du Sud. L'objectif principal de ce travail était d’évaluer le parcours de vie et de soin des femmes trans à Cayenne.

## Méthodes

Une étude épidémiologique transversale a été faite à Cayenne auprès des femmes trans. Un questionnaire de 130 questions a été administré avec une consultation de dermatologie et de prévention puis une orientation selon les problématiques soulevées. Résultats. Au total, seules 17 femmes trans ont pu être identifiées et incluses, âgées en moyenne de 29 ans. 53% étaient infectées par le VIH et sous TARV. Elles vivaient en Guyane depuis 2 ans en médiane et étaient d'origines variées: brésilienne (n = 5), guyanienne (n = 5), haïtienne (n = 5), cubaine (n = 1) et française (n = 1). Elles rapportaient une précarité du logement avec 83 *%* ayant déjà vécu en squat et une précarité alimentaire, 70% ayant passé une journée entière sans manger faute d'argent dans le mois passé. Leur premier rapport sexuel avait eu lieu en moyenne à 13 ans. Un antécédent d'agression sexuelle était très majoritaire (94%) ainsi que de viol (70%). Le recours au TDS concernait l'ensemble des femmes trans, au moins une fois dans leur vie. L'usage systématique du préservatif était rapporté dans 87,5% des cas avec les clients et 59% avec leur partenaire régulier. Le niveau de connaissance sur les traitements prévenant l'infection à VIH était bas. La prophylaxie pré-exposition (PrEP) était inconnue dans 23,5% des cas, et le traitement post-exposition dans 41% des cas. 18% étaient intéressées et ont été orientées en consultation PrEP. Les violences physiques et psychologiques étaient très fréquemment rapportées (100% et 94%), et 53% avaient déjà pensé à faire une tentative de suicide.

## Discussion et conclusion

Les femmes trans en Guyane sont précaires, victimes de toutes les formes de violences et éloignées du soin. Le risque de VIH est important et aggravé par la méconnaissance des traitements préventifs, la pratique du TDS ainsi que les violences physiques et sexuelles subies. Le parcours de transition sur place doit être développé, et un renforcement des actions de prévention en « aller vers » avec une base communautaire est nécessaire.

# Research article: Causes des adénopathies chez les patients vivant avec le VIH en Guyane: épidémiologie et comparaison

**Published:** 2023-10-31

**Authors:** Morgane BOURNE-WATRIN, Kinan DRAK ALSIBAI, Antoine ADENIS, Sophie BARON, Dufens PIERRE-LOUIS, Loïc EPELBOIN, Mathieu NACHER, Félix DJOSSOU, Pierre COUPPIÉ

**Affiliations:** 1 Service de dermatologie-vénérologie, Centre hospitalier de Cayenne, Cayenne, Guyane; 2 Laboratoire d'anatomie et cytologie pathologiques, Centre hospitalier de Cayenne; 3 Centre d'investigation clinique Antilles-Guyane, CIC INSERM 1424, Centre hospitalier de Cayenne; 4 Tropical Biome & Immunophysiopathology (TBIP), Université de Guyane, Université de Lille, CNRS UMR9017, INSERM U1019, Institut Pasteur de Lille, Centre d'infection et d'immunité de Lille (CIIL), France; 5 Institut Pasteur de la Guyane, Cayenne; 6 Service de médecine A, Centre hospitalier de Cayenne; 7 Unité des maladies infectieuses et tropicales, Centre hospitalier de Cayenne

## Introduction

Les adénopathies peuvent être présentes à tous les stades de l'infection par le VIH, réactionnelles à l'infection ellemême ou d'origine maligne néoplasique ou infectieuse. L'objectif de notre étude était de décrire les causes d'adénopathies chez les personnes vivant avec le VIH (PvVIH) en Guyane française (GF) et de les comparer entre elles.

## Méthodes

Nous avons réalisé une étude transversale rétrospective descriptive multicentrique entre janvier 2005 et décembre 2021. La population d'inclusion correspondait aux PvVIH qui avaient bénéficié d'une cytoponction ou d'une biopsie ganglionnaire ayant été analysée dans le service d'anatomopathologie de Cayenne.

## Résultats

152 adultes ont été inclus. Le sexratio M/F était de 1,6 et l’âge médian de 43 [35-51] ans. La maladie responsable de l'adénopathie était disséminée chez 51% et représentait l’évènement classant SIDA chez 43%. Le nombre médian de CD4 était de 185/mm^3^ [60-344]. Les principales étiologies étaient: histoplasmose (25%), tuberculose (24%), lymphadénite réactionnelle (22%), syndrome lymphoprolifératif et tumeurs solides (11% chacun). Des causes multiples étaient retrouvées dans 5%. Les adénopathies présentes depuis < 1 mois étaient retrouvées dans 60% des cas de tuberculose et 66% d'histoplasmose, jamais dans les adénites réactionnelles. La tuberculose pouvait être retrouvée indépendamment du nombre de CD4, l'histoplasmose était presque toujours retrouvée < 500 CD4/mm^3^, les métastases jamais < 100 CD4/mm^3^. La tuberculose était la seule cause d'adénopathies fistulisées ou fluctuantes (18% chacune) et la première cause de nécrose radiologique (33%). Les marqueurs de diagnostic indirects tels que ß-2-microglobuline, ß-D-glucane, galactomannane, sérologie histoplasmose et quantiferon ne permettaient pas d'orientation diagnostique.

## Discussion et conclusion

Au cours des 15 dernières années et dans le cadre de l'objectif 90-90-90 de l'ONUSIDA, les infections opportunistes représentaient 55% des causes d'adénopathies. L'histoplasmose, déjà reconnue comme première affection classant SIDA et première cause de décès liée au SIDA, est également la première cause d'adénopathie chez les PvVIH en GF.

# Research article: Probable anaplasmose chez un randonneur en Guyane: pourquoi il faut continuer à rechercher les maladies bactériennes transmises par les tiques

**Published:** 2023-10-31

**Authors:** Morgane BOURNE-WATRIN, Kinan DRAK ALSIBAI, Olivier DURON, Justin DESTOOP, Félix DJOSSOU, Loïc EPELBOIN, Pierre COUPPIÉ

**Affiliations:** 1 Service de dermatologie-vénérologie, Centre hospitalier de Cayenne, Guyane; 2 Laboratoire d'anatomie et cytologie pathologiques, Centre hospitalier de Cayenne; 3 MIVEGEC (Maladies Infectieuses et Vecteurs: Écologie, Génétique, Évolution et Contrôle), Centre national de la recherche scientifique (CNRS), Institut pour la recherche et le développement (IRD), Université de Montpellier (UM), Montpellier, Hérault; 4 Unité des maladies infectieuses et tropicales, Centre hospitalier de Cayenne; 5 Tropical Biome & Immunophysiopathology (TBIP), Université de Guyane, Université de Lille, CNRS UMR9017, INSERM U1019, Institut Pasteur de Lille, Centre d'infection et d'immunité de Lille (CIIL)

## Contexte

Les tiques sont les deuxièmes vecteurs les plus fréquents de maladies infectieuses humaines dans le monde, après les moustiques. Peu de données sont disponibles sur les maladies transmises par les tiques en Guyane française (GF), mais les données brésiliennes décrivent plusieurs espèces de rickettsies comme pathogènes. De nouvelles espèces de Borrelia, Rickettsia et Anaplasma ont récemment été détectées en GF.

## Cas

Un homme de 33 ans consultait pour fièvre, myalgies, rash, escarres d'inoculation aux membres inférieurs 10 jours après avoir été piqué par des centaines de tiques lors d'une randonnée en forêt profonde, près de Saül. La biologie retrouvait une polynucléose neutrophile à 7,9 G/L, une lymphopénie modérée à 1,4 G/L sans anémie ni thrombopénie, une cytolyse (ASAT5N, ALAT 2N), une cholestase (GGT7N, PAL 1,5N) et une CRP à 78 mg/L avec une créatinine et des CPK normaux. Les sérologies VIH, VHA, VHB, VHC, VHE, CMV, syphilis, fièvre Q, l'antigène NS1, les PCR dengue et leptospirose étaient négatifs. La biopsie cutanée retrouvait un infiltrat inflammatoire dermique polymorphe associant lymphocytes, polynucléaires éosinophiles et macrophages en périvasculaire et péri-annexielle avec un bacille gram-négatif intramacrophagique. Les sérologies *Rickettsia conorii* et *typhi* étaient négatives mais positives pour *Anaplasma phagocytophilum* en IgM et IgG. Les PCR sanguines Anaplasma, Borrellia, Rickettsia, Babesia étaient négatives, de même que la PCR cutanée à Anaplasma. L’état du patient s'est amélioré sous doxycycline et amoxicilline/clavulanate (cellulite associée).

## Conclusions

Même si non prouvée par la biologie moléculaire, cette infection à bactérie gram-négative transmise par les tiques souligne le fait que très peu de choses sont connues sur ces zoonoses en GF. Dans cet écosystème particulier où le diagnostic de nouvelles espèces est difficile avec des outils moléculaires non conçus pour détecter des pathogènes différents des espèces connues, les cliniciens devraient être conscients de ces risques de zoonoses.

# Research article: Maladie de Lyme en Guyane: étude descriptive et qualitative des patients suivis pour une suspicion de maladie de Lyme au Centre hospitalier de Cayenne entre 2010 et 2022

**Published:** 2023-10-31

**Authors:** Tom CARTAU, Marc-Alexandre TAREAU, Anaïs ESKENAZI, Gaëlle WALTER, Catherine COIGNARD, Ward SCHROOTEN, Félix DJOSSOU, Loïc EPELBOIN

**Affiliations:** 1 Unité des maladies infectieuses et tropicales, Centre hospitalier Andrée Rosemon, Cayenne; 2 Laboratoire Écologie Évolution Interactions des Systèmes amazoniens, Université de Guyane, Cayenne; 3 Unité de médecine interne, Centre hospitalier de Cayenne; 4 Unité des maladies infectieuses et tropicales, Centre hospitalier de Cayenne; 5 Biologiste médicale, Laboratoire Eurofins Biomnis, Ivry-sur-Seine, Val-de-Marne; 6 Département d'information médicale, Centre hospitalier de Cayenne; 7 Unité des maladies infectieuses et tropicales, Centre hospitalier de Cayenne

## Introduction

La maladie de Lyme est une zoonose fréquente dans les régions tempérées, causée par un spirochète du genre *Borrelia burdorferi* sensu lato transmis par les tiques du genre *Ixodes.* Cette maladie est difficile à diagnostiquer du fait de nombreux symptômes aspécifiques, et est sujette à de nombreux débats. Sa présence en Amérique du Sud est discutée, et n'a jamais été étudiée en Guyane.

## Matériel et méthode

Nous avons réalisé un recueil rétrospectif des données des patients ayant été suivis pour une suspicion de maladie de Lyme en Guyane entre 2010 et 2022. Nous avons parallèlement interrogé ces patients afin d’évaluer leur parcours médical à l'aide d'un questionnaire pré-établi selon les recommandations COREQ.

## Résultats

Sur la période d’étude, 26 patients ont été inclus pour l'analyse descriptive. Ces patients étaient majoritairement des hommes (14/26, 54%) avec un âge médian de 49 ans [35,5-61]. Leur plainte principale était des symptômes rhumatologiques (23/26, 88%), suivis des symptômes neurologiques (16/26, 61%). Deux (8%) de ces patients étaient nés en Guyane française. Le diagnostic de maladie de Lyme a été confirmé chez 2/26 (8%) des patients, probable chez 2/26 (8%). Un diagnostic différentiel a été trouvé chez 14/26 (54%) des patients (4 maladies neurologiques ou psychiatriques dont une sclérose en plaques, 5 maladies infectieuses, 2 maladies rhumatologiques, 2 maladies systémiques, 1 fibromyalgie). Nous avons parallèlement interrogé 9 patients pour l’étude qualitative, mettant en évidence 4 thèmes principaux: un parcours long vers un diagnostic, des critiques vis-à-vis des tests et des traitements, l'impact de la maladie, et le besoin d'améliorer la prise en charge.

## Discussion

Cette étude est la première s'intéressant aux patients suivis pour une maladie de Lyme en Guyane. Les caractéristiques principales des patients étaient comparables aux études antérieures. Une patiente a présenté un érythème migrant objectivé après une morsure de tique en Guyane, cinq patients décrivent un érythème migrant en Guyane n'ayant pas été objectivé, mais le lieu d'inoculation est souvent difficile à déterminer. Les patients vivent fréquemment un long parcours avec des symptômes persistants et invalidants, qu'ils relient à la maladie de Lyme même si ce diagnostic n'est pas posé par un médecin. Cette conviction peut mener à des traitements abusifs, et à méconnaître un éventuel diagnostic différentiel.

# Research article: Étude descriptive du rôle infirmier spécifique dans un centre antirabique à forte activité

**Published:** 2023-10-31

**Authors:** Mylène CÈBE, Brigitte ROMAN-LAVERDURE, Marie FOUILLET, Dominique BELOEIL, Carlisi ROCCO, Loïc EPELBOIN, Félix DJOSSOU, Paul LE TURNIER

**Affiliations:** 1 Unité des maladies infectieuses et tropicales et Centre de traitement antirabique. Centre hospitalier de Cayenne, Guyane; 2 ARS Guyane

## Introduction

La rage est une zoonose virale dont la transmission se fait par morsure/griffure ou léchage d'une peau lésée. La Guyane est particulièrement exposée du fait de la présence endémique de rage due aux chauves-souris. Notre objectif était de décrire et d'analyser le rôle et les compétences spécifiques de l'infirmière (IDE) dans un Centre antirabique (CAR) avec une forte activité.

## Matériels et méthodes

Tout cas de risque d'exposition au virus de la rage est recensé au CAR de Cayenne. L'IDE assure le lien avec les Antennes antirabiques (AAR) situées sur le reste du territoire. Pendant 10 ans, la gestion du CAR a été assurée par un seul effectif à temps plein. Depuis janvier 2022, une 2^e^ IDE est présente. Une analyse descriptive du rôle de l'IDE et des actions menées de janvier 2022 à janvier 2023 a été réalisée.

## Résultats

Durant la période d’étude, l'IDE du CAR avait pour mission de collecter les données liées à un risque d'exposition rabique afin d’évaluer la conduite à tenir (553 dossiers en 2022). Ce recueil lui permettait d’évaluer le besoin de mise en place d'une prophylaxie post-exposition (PPE). Cette PPE consiste à appliquer un schéma vaccinal, avec ou sans injection locale d'immunoglobulines (Ig). Après validation médicale, l'IDE a réalisé les soins locaux (pansements, injections d'Ig (128) et vaccins antirabiques (324). Elle a assuré la visite de 8 sites afin d'informer et de former le personnel des AAR. Elle a géré la formation au Centre hospitalier de Cayenne auprès des médecins de différents services et des médecins assurant les astreintes téléphoniques. Durant l'année 2022, l'IDE du CAR a mis en place le protocole IPC (Institut Pasteur du Cambodge) recommandé par l'OMS en 2018. Ce protocole réduit le temps nécessaire à une PPE complète et le nombre de flacons utilisés en vaccinant en intradermique.

## Conclusion

L'IDE du CAR permet de centraliser les expositions et d'adapter la prise en charge des personnes en fonction des situations. Elle assure de façon autonome le suivi de chaque dossier, les activités de soins et la gestion des formations. La poursuite des visites au sein du réseau antirabique et la formation des professionnels permettront d'optimiser le suivi des dossiers et de diminuer le nombre de PPE incomplètes.

# Research article: Prise en charge du risque d'exposition au virus de la rage en Centre antirabique et en Antennes antirabiques

**Published:** 2023-10-31

**Authors:** Mylène CÈBE, Brigitte ROMAN-LAVERDURE, Marie FOUILLET, Dominique BELOEIL, Carlisi ROCCO, Loïc EPELBOIN, Félix DJOSSOU, Paul LE TURNIER

**Affiliations:** 1 Unité des maladies infectieuses et tropicales, Centre hospitalier de Cayenne, Guyane; 2 ARS Guyane

## Introduction

Le Centre antirabique (CAR) de Cayenne est géré par une infirmière (IDE) qui, sous supervision médicale, centralise les avis, pose les indications de prophylaxie postexposition (PPE) et est la seule à pouvoir administrer les immunoglobulines antirabiques (IgAR) pour les RER (risques d'exposition au virus de la rage) de catégorie 3 (RER3). Les autres centres de soin sont des Antennes antirabiques (AAR) qui signalent leurs dossiers au CAR et assurent la vaccination sans personnel dédié. Notre objectif était de comparer les RER et leurs prises en charges selon le recours initial en CAR ou en AAR.

## Matériels et méthodes

L'intégralité des dossiers de RER a été revue, pour l'année 2022, à partir de la base de données du CAR où les informations sont colligées prospectivement. Une comparaison a été faite sur les expositions les plus à risque, les RER3. Ces expositions nécessitent une PPE complète (IgAR et vaccination AR). Les taux de RER validés, d'indication de PPE en cas de RER, d'initiation de PPE vaccinale (PPEv) et de complétude de PPE ont été comparés en fonction des groupes définis par le lieu de recours initial: CAR ou hors CAR. Les analyses bivariées ont été faites par test du Chi2 avec un seuil de significativité fixé à 0,05.

## Résultats

En 2022, 553 signalements ont été traités. Les RER3 étaient les plus fréquents avec 513 (94,3%) cas sans différence entre les groupes (94,8% *vs* 93,3%, p = 0,47). Pour les RER3, une PPE était indiquée dans 211 (41,1%) cas. L'indication était plus fréquente pour le groupe CAR (47,1% vs 30,2%, p < 0,001). Une PPE complète était indiquée pour 186 (88,2%) cas de RER3 sans différence entre les groupes (87,8% *vs* 89,1%, p = 0,80). Le taux d'administration d'IgAR était de 82,8% (93,4% *vs* 53,1%, p < 0,001). Au final, les cas du groupe AAR avec un RER3 avaient un risque relatif de PPE incomplète de 4,3 (IC95% 3,0-6,l).

## Conclusion

La prise en charge en zones éloignées du CAR était associée à moins d'indication mais à une moindre réalisation des PPE. L'absence de personnel dédié et les difficultés de déplacement vers Cayenne pourraient jouer un rôle dans la mise en place et la complétude d'une PPE. Il semble nécessaire de s'impliquer sur la prise en charge dans ces zones isolées, voire d'envisager une campagne de vaccination préventive.

# Research article: Accès palustre grave à Saül chez un patient revenant du Cameroun alors qu'il prenait une prophylaxie à base de tisane d’*Artemisia annua*

**Published:** 2023-10-31

**Authors:** Olivia COHEN, Mathilde BOUTROU, Mathieu NACHER, Éric CAUMES, Félix DJOSSOU, Loïc EPELBOIN

**Affiliations:** 1 Unité des maladies infectieuses et tropicales, Centre hospitalier de Cayenne Andrée Rosemon, Cayenne, Guyane; 2 Centre d'investigation clinique Antilles-Guyane, CIC INSERM 1424, Centre hospitalier de Cayenne Andrée Rosemon; 3 Service des maladies infectieuses et tropicales, Groupe hospitalier universitaire Pitié-Salpêtrière, Paris

## Introduction

Le paludisme est une maladie potentiellement mortelle présente dans les régions tropicales telles que l'Afrique subsaharienne et l'Amérique du Sud. Devant la forte prévalence en Afrique, il est recommandé aux voyageurs de prendre un traitement prophylactique. Récemment, des mouvements pour une médecine « plus naturelle » ont popularisé l'utilisation de tisanes d’*Artemisia* comme une alternative aux antipaludiques conventionnels. *Artemisia annua* est une plante médicinale contenant de l'artémisinine dont les dérivés sont utilisés dans les ACT (Artemisinin-based combination therapy), traitements de première intention du paludisme. Cependant, les preuves d'efficacité des tisanes d’*Artemisia* sont inexistantes et les doses d'artémisinine absorbées semblent aléatoires. Nous rapportons un cas d'accès palustre grave à Saül chez un patient revenant d'un voyage au Cameroun avec comme seule prophylaxie la prise régulière de tisane d’*Artemisia.*

## Résumé du cas

Un patient de 22 ans, retrouvé par les autorités en forêt à peine conscient, est ramené jusqu'au CDPS de Saül dans un état général très dégradé. Une recherche systématique de paludisme par TDR retrouve 2 bandes, Pf et PAN, suggérant un accès à *Plasmodium falciparum.* Devant la gravité clinique, un traitement par artéméther/luméfantrine est débuté et le patient est héliporté jusqu'au Centre hospitalier de Cayenne. Le bilan retrouve une parasitémie à 1,62% avec une insuffisance rénale aiguë (créatinine 325 pmol/L) sans autres critères d'accès palustre sévère. Après interrogatoire, il apparaît qu'il revient de 4 mois au Cameroun avec une « prophylaxie anti-palustre » basée uniquement sur des tisanes d’*Artemisia.* Ses symptômes ont débuté plusieurs jours auparavant et il a consulté à Kourou mais a préféré continuer la tisane plutôt que le traitement prescrit. Hospitalisé, le patient s'améliore progressivement et rentre à domicile avec une fonction rénale restant anormale (créatinine 122 pmol/L) à J5. Il reste persuadé de l'efficacité de la tisane.

## Discussion et conclusion

La tisane d’*Artemisia* est vendue sur les marchés et sur Internet comme une alternative fiable aux antipaludiques sans aucune preuve d'efficacité. Sa consommation expose les voyageurs à des doses sub-thérapeutiques d'artémisinine ne les protégeant pas mais pouvant favoriser la propagation de résistance à l'artémisinine.

# Research article: Cinétique de l'activité de l'enzyme Glucose-6-Phosphate Déshydrogénase (G6PD) au cours des accès palustres à *Plasmodium vivax* en Guyane

**Published:** 2023-10-31

**Authors:** Laureen DAHURON, Juste GOUNGOUNGA, Moustapha DRAME, Maylis DOUINE, Mathieu NACHER, Théo BLAISE, Émilie MOSNIER, Lise MUSSET, Félix DJOSSOU, Loïc EPELBOIN

**Affiliations:** 1 Unité des maladies infectieuses et tropicales, Centre hospitalier de Cayenne Andrée Rosemon, Cayenne, Guyane; 2 Université de Bourgogne, Registre bourguignon des Cancers digestifs, Dijon, Côte-d'Or; 3 INSERM, UMR1231, Dijon; 4 Service de recherche clinique et d'innovation, Centre hospitalier de Fort-de-France, Martinique; 5 Centre d'investigation clinique Antilles-Guyane, CIC INSERM 1424, Centre hospitalier de Cayenne Andrée Rosemon; 6 INSERM, IRD, SESSTIM (Sciences économiques et sociales de la santé et traitement de l'information médicale), ISSPAM, Université Aix-Marseille, Marseille, Bouches-du-Rhône; 7 Université des Sciences de la santé /Agence nationale de recherche sur le Sida, Phnom Penh, Cambodge; 8 Laboratoire de parasitologie, Centre national de référence du paludisme, Institut Pasteur de la Guyane, Cayenne

## Introduction

*Plasmodium vivax* est responsable de récurrences par la formation d'hypnozoïtes hépatiques. Pour les éviter, un traitement radical par primaquine est utilisé. Celui-ci est contre-indiqué en cas de déficit en Glucose-6-Phosphate-Déshydrogénase (G6PD). En Guyane, l'activité de la G6PD est dosée à J14 pour éviter de méconnaître des patients déficitaires en G6PD en contexte hémolytique. Le but de cette étude est d’évaluer la cinétique de la G6PD au cours d'un accès palustre et les facteurs associés à ses variations.

## Méthodes

C’était une étude monocentrique et rétrospective. Nous avons analysé l'activité de la G6PD entre J1 et J28 chez des patients traités pour une infection à *P. vivax* par chloroquine ou dérivé de l'artémisinine au Centre hospitalier de Cayenne entre janvier 2018 et décembre 2020. Les patients ont été répartis dans trois bras selon le nombre de G6PD disponible au cours de l'accès palustre étudié. Le « groupe global » inclut les bras 1, 2 et 3.

## Résultats

L'activité de la G6PD varie significativement au cours du temps (p = 0,004) et se stabilise à partir du troisième jour. Aucun patient déficitaire en G6PD n'a eu de dosages faussement normaux en phase aiguë de l'accès palustre. Le sexe et le taux de réticulocytes sont statistiquement associés aux variations de la G6PD. 26,5% des patients ont été perdus de vue avant traitement par primaquine. Sur les 223 patients inclus dans le groupe global, aucun ne présentait un déficit en G6PD sévère et seulement 3 (1,35% ± 0,78) présentaient un déficit entre 10 et 30%.

## Discussion et conclusion

L'activité de la G6PD varie au cours du temps lors d'un accès palustre à *P. vivax*. Le dosage de la G6PD du troisième jour peut devenir la valeur de référence pour délivrer la primaquine plus tôt afin d'optimiser le traitement radical. Ceci permettrait notamment de diminuer les récurrences précoces et le nombre de perdus de vue.

# Research article: Caractéristiques et prise en charge des patients adultes admis au Centre hospitalier de Cayenne de 2015 à 2017 en lien avec une infection à virus Zika prouvée ou suspectée

**Published:** 2023-10-31

**Authors:** Clément DECROCQ, Christelle PRINCE, Rémi MUTRICY, Jean-Michel CAUVIN, Ward SCHROOTEN, Félix DJOSSOU, Loïc EPELBOIN, Paul LE TURNIER

**Affiliations:** 1 Centre hospitalier universitaire Amiens-Picardie, Somme; 2 Infirmière IDE régionale hospitalière de santé publique, Unité des maladies infectieuses et tropicales (UMIT), ARS, SPF, Centre hospitalier de Cayenne, Guyane; 3 SAU SAMU SMUR, Centre hospitalier de Cayenne; 4 Département d'information médicale, Centre hospitalier de Cayenne; 5 Unité des maladies infectieuses et tropicales, Centre hospitalier de Cayenne

## Introduction

L'infection à virus Zika (ZIKV) est une arbovirose qui a provoqué de décembre 2015 à mars 2017 une première épidémie en Guyane. L'objectif était de décrire les caractéristiques cliniques et les modalités diagnostiques des patients admis à l'hôpital de Cayenne entre décembre 2015 et mars 2017 pour infection ZIKV prouvée ou suspectée et à évaluer le coût de leur prise en charge.

## Méthodes

Dans cette étude monocentrique rétrospective, les patients admis au Centre hospitalier de Cayenne pour diagnostic d'arbovirose entre décembre 2015 et mars 2017 ont été analysés à partir des codages de séjour aux urgences et en hospitalisation. Les critères d'inclusion étaient un âge > 15 ans, l'absence de grossesse et avoir au moins 3 symptômes évocateurs. Un cas était confirmé si la RT-PCR ZIKV était positive, probable en cas de détection d'IgM anti-ZIKV, possible si le ZIKV n’était pas testé mais avec exclusion d'autres arboviroses, suspect en l'absence d'examen biologique. Étaient exclus les patients avec une RT-PCR ZIKV négative. Les caractéristiques des cas suspects étaient comparées aux autres (confirmés, probables et possibles réunis). Les coûts d'hospitalisation et des consultations initiales et de suivi ont été recueillis. Une non-opposition a été obtenue.

## Résultats

Parmi les 347 patients identifiés, 130 patients ont été inclus après exclusion principalement des enfants et femmes enceintes. Le sex-ratio F/H était de 2,2. Les cas étaient confirmés/probables/possibles/suspects pour 9 (6,9%), 4 (3,1%), 23 (17,7%) et 94 (72,3%) patients respectivement. Les cas suspects étaient significativement plus jeunes, moins comorbides, consultaient plus tôt, avaient plus souvent une éruption ou une conjonctivite et moins souvent de douleurs abdominales. Onze (8,5%) patients ont été hospitalisés dont 6 pour une complication neurologique sévère. La durée médiane d'hospitalisation était de 5 jours. Aucun décès n'a été rapporté. Le coût total engendré par les hospitalisations et l'ensemble des consultations était de 140 411 €.

## Discussion et conclusion

La majorité des patients inclus dans l’étude avaient une forme bénigne ambulatoire. La présence fréquente de signes très évocateurs (éruption et conjonctivite) en période épidémique pouvait expliquer un moindre recours au diagnostic biologique. Cette étude est limitée par la méthodologie d'identification des cas mais suggère que la charge en soins sur l'hôpital de Cayenne liée à cette première épidémie de Zika était modérée.

# Research article: Dépistage et prévalence des infections sexuellement transmissibles en milieu carcéral guyanais français de 2013 à 2020: un état des lieux à l'Unité sanitaire en milieu pénitentiaire de Rémire

**Published:** 2023-10-31

**Authors:** Stella DENOEL, Aude LUCARELLI, Marie DANIEL, Vanessa SCHIEMSKY, Sébastien RABIER, Évelyne VIERENDEELS, Antoine ADENIS, Mathieu NACHER, Timothée BONIFAY

**Affiliations:** 1 Unité sanitaire en milieu pénitentiaire de Rémire, Centre hospitalier Andrée Rosemon, Cayenne, Guyane; 2 Département universitaire de médecine générale, Université des Antilles, Pointe-à-Pitre, Guadeloupe; 3 Comité régional de lutte contre le VIH, Centre hospitalier Andrée Rosemon, Cayenne; 4 Centre d'Investigation clinique – Épidémiologie clinique, Centre hospitalier Andrée Rosemon, Cayenne

## Introduction

Les infections sexuellement transmissibles (IST) sont un enjeu majeur de santé publique dans le monde. La situation de la Guyane qui présente la prévalence du VIH la plus élevée de France est préoccupante. Or, les personnes détenues qui font partie des populations marginalisées sont plus à risque de contracter des infections sexuellement transmissibles.

## Méthodes

L'objectif principal de notre étude était d'estimer les prévalences des IST à l'entrée au Centre pénitentiaire de Guyane française. Une étude monocentrique, transversale, rétrospective a été menée à l'Unité sanitaire en milieu pénitentiaire de Rémire pour la période allant du 1^er^ janvier 2013 au 31 décembre 2020. La population étudiée était composée des personnes entrantes. Les résultats des sérologies sanguines et des PCR urinaires ont été analysés.

## Résultats

Sur les 7115 personnes entrantes, 4140 personnes (58,9%) ont bénéficié du dépistage d'entrée au Centre pénitentiaire de Guyane (CPG). Les prévalences des infections sexuellement transmissibles étaient de 8,3% (IC95%: 6,0-11,0) pour la chlamydia, de 3,1% (IC95%: 2,7-3,6) pour le VIH, de 2,1% (IC95%: 1,8-2,5) pour le VHB, de 0,8% (IC95%: 0,2-2,0) pour la syphilis, de 0,7% (IC95%: 0,4-1,0) pour le gonocoque et de 0,4% (IC95%: 0,2-0,5) pour le VHC.

## Discussion et conclusion

Le biais principal rencontré dans notre étude était le biais d'information. Le taux de dépistage à l'entrée au CPG était dans la moyenne nationale. Il pourrait être amélioré par le déploiement des Tests rapides d'orientation diagnostique (TROD). Les prévalences des infections sexuellement transmissibles étaient supérieures à celles observées dans la population générale de Guyane pour la chlamydia, le VIH et le VHB. L'incarcération devrait donc être une opportunité de dépister et traiter les détenus porteurs d'IST en vue d'améliorer le pronostic individuel et collectif en évitant la dissémination de ces dernières à la libération.

# Research article: Comparaison des cas humains de leptospirose entre la Guyane, Mayotte et La Réunion de 2018 à 2021

**Published:** 2023-10-31

**Authors:** Anissa DESMOULIN, Olivier MAILLARD, Renaud BLONDÉ, Louis COLLET, Pablo TORTOSA, Félix DJOSSOU, Paul LE TURNIER, Marie-Christine JAFFAR-BANDJEE, Anne OVIZE, Loïc RAFFRAY, Loïc EPELBOIN

**Affiliations:** 1 Unité des maladies infectieuses et tropicales, Centre hospitalier de Cayenne, Guyane; 2 Centre hospitalier universitaire de La Réunion, Saint-Denis, La Réunion; 3 Centre hospitalier de Mayotte, Mamoudzou, Mayotte; 4 Centre d'investigation clinique, CIC INSERM 1410, CHU Sud Réunion, Saint-Pierre, La Réunion; 5 Université de La Réunion, CNRS 9192, INSERM U1187, IRD 249, UMR PIMIT, CHU de La Réunion, Unité mixte Processus infectieux en milieu insulaire tropical (PIMIT), Plateforme technologique CYROI, Sainte-Clotilde, La Réunion; 6 Département de recherche, innovation et santé publique (DRISP), Centre hospitalier de Cayenne, Guyane; 7 Laboratoire Eurofins Biomnis, Lyon, Rhône

## Introduction

La leptospirose est une zoonose causée par les bactéries du genre *Leptospira.* Peu de données existent évaluant la présentation clinique et la sévérité des cas humains. La Guyane, Mayotte et La Réunion sont trois départements d'outre-mer français dont les incidences sont parmi les plus élevées de France. L'objectif de ce travail était de comparer la sévérité des cas humains de leptospirose de ces trois départements.

## Méthodes

Il s'agit d'une étude rétrospective multicentrique incluant tous les patients âgés de plus de 18 ans avec une leptospirose prouvée par PCR sur la période de janvier 2018 à décembre 2021 au Centre hospitalier de Cayenne et de janvier 2018 à mai 2020 à Mayotte et à La Réunion. Les données démographiques, cliniques et biologiques ont été recueillies sur ces périodes. Le critère de jugement principal était la sévérité définie par: le recours à la ventilation mécanique, à l’épuration extra-rénale, aux amines vasoactives et/ou le décès.

## Résultats

Au total 560 patients ont été inclus (222 à Mayotte, 268 à La Réunion et 70 à Cayenne). Les patients étaient significativement plus jeunes à Mayotte (35 ans +/- 12,1) qu’à La Réunion et qu’à Cayenne (48,3 +/- 15,8 et 42 ans +/- 15,1), p < 0,001. Les cas étaient représentés majoritairement par des hommes dans les trois départements. Le contact avec des rats au domicile était plus fréquemment rapporté à Mayotte (59%, n = 23) et à Cayenne (76%, n = 35). À La Réunion, la voie d'exposition la plus fréquente était lors des activités professionnelles (47%, n = 86). Il existait moins de formes graves à Mayotte (8%, n = 18) qu’à La Réunion (24%, n = 65) ou qu’à Cayenne (24%, n = 17), p < 0,001. Parmi les patients hospitalisés, l'atteinte rénale était la plus fréquente à Mayotte (9%, n = 9) et à La Réunion (22%, n = 48), alors qu'il s'agissait de l'atteinte respiratoire à Cayenne (15%, n = 9). Les létalités observées étaient respectivement de 2,0% (n = 2), 1,9% (n = 4) et 2,9% (n = 2).

## Discussion et conclusion

La leptospirose reste un problème majeur de santé publique, notamment dans les départements de la Guyane, de Mayotte et de La Réunion. Cependant, des différences peuvent être observées dans ces trois zones, tant sur la population qu'elle touche, que sur les facteurs d'exposition et sa sévérité. Les formes de leptospirose en Guyane et à La Réunion nécessitent plus souvent un recours aux traitements de suppléances qu’à Mayotte. En revanche la létalité observée est comparable dans ces trois départements. Des études futures seraient nécessaires afin de déterminer le rôle potentiel des espèces bactériennes en cause.

# Research article: Premier cas d'angiostrongylose abdominale à *Angiostrongylus costaricensis* en Amazonie française

**Published:** 2023-10-31

**Authors:** Anissa DESMOULIN, Alessia MELZANI, Céline DARD, Mathieu NACHER, Félix DJOSSOU, Mohamed Kinan DRAK ALSIBAI, Loïc EPELBOIN

**Affiliations:** 1 Service des maladies infectieuses et tropicales, Centre hospitalier de Cayenne, Guyane; 2 Laboratoire Human Leukocyte Antigen (HLA), Établissement français du sang (EFS), La Tronche, Isère; 3 Laboratoire d'anatomopathologie, Centre hospitalier de Cayenne; 4 Centre d'investigation clinique Antilles-Guyane, INSERM 1424, Centre hospitalier de Cayenne

## Introduction

L'angiostrongylose abdominale est une maladie parasitaire causée par *Angiostrongylus costaricensis* au stade larvaire (L3) dont le premier cas a été décrit au Costa Rica par Pedro Morera et Rodolfo Cespedes en 1971. Elle a ensuite été rapportée du Texas au sud de l'Argentine, y compris les régions amazoniennes et les Caraïbes. Rares sont les cas identifiés en Amazonie et aucun cas n'a été notifié sur le Bouclier des Guyanes.

## Présentation du cas

Nous présentons le cas d'un homme de 34 ans qui a vécu pendant 7 ans en Guyane et séjourné entre 2018 et 2020 en Guadeloupe (10 jours), Martinique (3 jours) et île de Tobago (21 jours). Il faisait fréquemment des randonnées dans la forêt amazonienne et consommait souvent du poisson cru pêché en rivière. Il a consulté en 2020 à l'hôpital de Cayenne pour des douleurs abdominales, des diarrhées récurrentes et des céphalées évoluant depuis 15 jours. L'hémogramme a montré une leucocytose avec une éosinophilie à 10,9 G/L (54,8% des leucocytes totaux) et une cytolyse hépatique avec ALAT 162 UI/L et ASAT 84 UI/L. Les sérologies *Taeniasolium, Entamoeba histolytica, Schistosoma* spp, *Toxocara canis, Trichinella spiralis* et *Strongyloides stercoralis* étaient négatives. La recherche de filaires et la sérologie *Ascaris lumbricoides* étaient positives en ELISA. La sérologie *dEchinoccoccus granulosus* était positive en ELISA mais non confirmée par le Western blot. La sérologie *Angiostrongylus* spp était revenue positive par Western blot. Une coloscopie a été réalisée et l'examen anatomopathologique du côlon montrait un infiltrat éosinophile avec des œufs ainsi qu'un agent intravasculaire allongé très évocateur d'angiostrongylose abdominale. Le patient a été traité par albendazole 400 mg une fois par jour pendant 14 jours associé à une dose unique d'ivermectine 200 pg/kg. Après un an de suivi, la symptomatologie s’était amendée et l’éosinophilie avait diminué à 1,5 G/L (14,8%).

## Discussion et conclusion

Il s'agit du premier cas décrit dans la partie orientale de l'Amazonie. Quelques cas ont été rapportés dans les régions amazoniennes de la Colombie, de l’Équateur et du Pérou. Dans le cas présent, le lieu de contamination de ce voyageur français est probablement la Guyane, mais la Guadeloupe, la Martinique et Tobago ne sont pas à exclure. Les hôtes définitifs *Rattus rattus* et *Rattus norvegicus* sont présents sur le Bouclier des Guyanes, rendant possible la transmission humaine. Des études complémentaires sont nécessaires pour déterminer la présence d'A. *costaricensis* chez les rongeurs du Bouclier des Guyanes.

# Research article: Covid-19 et vaccination: connaissances, attitudes et pratiques des personnes travaillant sur les sites d'orpaillage illégaux en Guyane

**Published:** 2023-10-31

**Authors:** Pierre DURAND, Célia BASURKO, Stephen VREDEN, Mathieu NACHER, Maylis DOUINE

**Affiliations:** 1 Centre d'investigation clinique Antilles-Guyane, CIC INSERM 1424, DRISP, Centre hospitalier de Cayenne, Guyane; 2 Département universitaire de médecine générale, Université des Antilles, Pointe-à-Pitre, Guadeloupe; 3 Médecine interne et maladies infectieuses, Hôpital académique de Paramaribo, Suriname; 4 Fondation pour l'avancement de la recherche scientifique au Suriname (SWOS), Paramaribo, Suriname

## Introduction

La Guyane est un département français frontalier du Brésil et du Suriname. Malgré le début de la vaccination anti-Covid début 2021, la pandémie de Covid-19 a fortement mis en tension le système de santé guyanais. Les personnes travaillant sur les sites d'orpaillage illégaux en Guyane, appelées *garimpeiros,* sont principalement d'origine brésilienne. Leurs conditions sanitaires sont précaires et l’étude de leur santé est difficile. Notre objectif était de décrire leurs connaissances, attitudes et pratiques vis-à-vis de la Covid-19 et de la vaccination.

## Méthodes

Nous avons mené une enquête transversale multicentrique internationale entre le 1^er^ mars et le 30 avril 2022 en Guyane et au Suriname, à l'aide d'un questionnaire.

## Résultats

Quatre-vingts personnes ont été incluses dont 95,0% étaient brésiliennes. La plupart avaient de bonnes connaissances sur la Covid-19. L'antibioprophylaxie avait été pratiquée par 10,0% des participants. Quarante-trois personnes pensaient avoir été infectées par la Covid-19 (53,8%). L'automédication était importante. Une majorité (62,5%) déclarait être vaccinée. Les personnes ayant déjà fait un test étaient les plus susceptibles d’être vaccinées (OR = 1,98, p = 0,009).

## Discussion et conclusion

Cette étude a été menée dans un contexte sanitaire exceptionnel avec un nombre limité de sujets inclus. La population était comparable à celle des études antérieures. Certaines attitudes et pratiques rappelaient celles observées au Brésil et le taux de vaccination était supérieur à celui de la population guyanaise.

## Conclusions

Les *garimpeiros* ont un bon niveau de connaissance sur la Covid-19. Cependant, plusieurs facteurs les amènent à avoir des pratiques négatives même si leur adhésion vaccinale est plus importante que celle de la population guyanaise. Les personnes les plus vaccinées étaient celles qui avaient déjà fait un test Covid, suggérant que ces personnes avaient été plus confrontées à la maladie, étaient plus soucieuses de leur santé ou avaient plus facilement accès aux centres de santé.

# Research article: Diagnostic anténatal et culture saramaca

**Published:** 2023-10-31

**Authors:** Marianne FRANCESCHI, Nadia THOMAS

**Affiliations:** 1 Sage-femme échographiste, Service de gynécologie et obstétrique, Centre hospitalier de Kourou; 2 Coordinatrice gynéco-obstétricale, Centres délocalisés de prévention et de soins (CDPS), Centre hospitalier de Cayenne

## Introduction

La crainte des parturientes d'origine saramaca d'une prise en charge dans le cadre du diagnostic anténatal s'explique-t-elle par une mauvaise communication entre soignants et soignées, ou par l'influence de la culture saramaca?

Le droit à l'information et le respect des profils individuels obligent les soignants, d'où la nécessité de fluidifier les rapports entre soignants et soignées pour une meilleure prise en charge dans le respect de l'individualité des femmes d'origine saramaca.

## Méthodologie

Étude descriptive avec entretiens semi-dirigés de 40 minutes à 1 heure de 42 parturientes ayant pour langue maternelle le saramaca, ceci de janvier à mai 2022.

## Principaux résultats

- Culture et méfiance, culture du secret: on ne parle pas du futur bébé ou seulement avec les très proches pour 86%.
- Première consultation tardive: 21% des femmes interrogées ont « peur des mauvaises intentions ».
- Culture et rituels:
- Césarienne: 56% de refus, car ensuite pas de bains rituels possibles.
- Infertilité: 37% d'acceptation de l'infertilité après médecine traditionnelle.
- Culture et nature: 1^re^ visite -si tout va bien, pas besoin de consultation.
- Priorité à la médecine traditionnelle (bains, tisanes et appel aux esprits) puis médecine occidentale si besoin.
- Non-maîtrise du français: 21% de visites tardives ne maîtrisent pas le français. Compréhension difficile lors d'explications pour examens et connaissances erronées: « avec échographie, on voit tout ». Pas d'informations précises sur les visites prénatales obligatoires.

## Conclusion

La culture saramaca influe sur la prise en charge des parturientes. Mais cette influence n'a pas de contours stricts et se veut discrète: on note une mosaïque choisie de soins. Avec une information et des outils adaptés, il est possible d'améliorer la prise en charge en respectant la culture et l'histoire de la femme.

# Research article: Développement d'un score pronostique de la mortalité de l'histoplasmose associée au VIH

**Published:** 2023-10-31

**Authors:** Ugo FRANÇOISE, Mathieu NACHER, Morgane BOURNE-WATRIN, Loïc EPELBOIN, Camille THOREY, Magalie DEMAR, Jean-François CAROD, Félix DJOSSOU, Pierre COUPPIÉ, Antoine ADENIS

**Affiliations:** 1 Centre d'investigation clinique Antilles-Guyane, CIC INSERM 1424, Centre hospitalier de Cayenne, Guyane; 2 DFR Santé, Université de Guyane, Cayenne; 3 Service de dermatologie, Centre hospitalier de Cayenne; 4 Unité des maladies infectieuses et tropicales, Centre hospitalier de Cayenne; 5 Service de médecine polyvalente, Centre hospitalier de l'Ouest guyanais, Saint-Laurent-du-Maroni, Guyane; 6 Laboratoire, Centre hospitalier de Cayenne; 7 Laboratoire, Centre hospitalier de l'Ouest guyanais, Saint-Laurent-du-Maroni

## Contexte

Le poids de l'histoplasmose est au moins aussi important que celui de la tuberculose en Amérique latine et la mortalité attribuable est encore plus élevée. L’évaluation de la gravité de la maladie, difficile, en est une des raisons.

## Méthodes

À partir de la base de données VIH-Histoplasmose de Guyane, nous avons tenté d'identifier les facteurs associés au décès à 30 jours après l'initiation des antifongiques et construit un score pronostique à partir d'un modèle de régression logistique multivariée. Nous avons évalué sa calibration par méthode graphique et sa performance de discrimination par courbe de Receiver Operating Characteristic (ROC), en utilisant les méthodes de rééchantillonnage et de validation croisée.

## Résultats

Sur les 415 patients inclus, 56 (13,5%) sont décédés dans les 30 premiers jours de traitement. Les facteurs associés au décès étaient un « Performance Status » ≥ 3, une altération de la vigilance, une dyspnée, une concentration de CRP ≥ 75 mg/L, un taux d'hémoglobine < 9 g/dL et/ou une numération plaquettaire < 100 000/mL, et un infiltrat interstitiel à la radiographie thoracique. Nous avons construit l’« Histoplasmosis case-Fatality Score » (HFS), un score pronostique en 12 points. Celui-ci était correctement calibré: plus le score est élevé, plus la proportion de décès précoces est importante. Un seuil ± 5 a permis de classer les patients comme vivants ou morts à 30 jours avec une précision de 81% (IC95% = 77-85), une sensibilité de 84% (IC95% = 73-93), une spécificité de 81% (IC95% = 76-84), une valeur prédictive positive de 40% (IC95% = 33-49) et une valeur prédictive négative de 97% (IC95% = 95-99). L’« Aera Under Curve » des courbes ROC des différentes méthodes de validation était stable entre 0,88 et 0,93.

## Conclusions

L'Histoplasmosis case-Fatality Score, dont la réalisation est facile et peu coûteuse, est le premier score de pronostic de l'histoplasmose associée au VIH. Le HFS a une bonne performance de classification pour discriminer les patients VIH avec histoplasmose disséminée à risque de décès dans les 30 jours de prise en charge thérapeutique. Après validation externe, il pourrait être un outil approprié pour évaluer la gravité et guider le choix du traitement d'induction.

# Research article: Profil des usagers d'une consultation hospitalière de médecine tropicale en Amazonie française: des vulnérabilités spécifiques?

**Published:** 2023-10-31

**Authors:** Marie FOUILLET, Elodie MATTHEW, Mylène CÈBE, Richard NALDJINAN-KODBAYE, Dominique BELOEIL, Félix DJOSSOU, Nicolas VIGNIER, Mathilde BOUTROU, Guillaume ICHER, Paul LE TURNIER, Loïc EPELBOIN

**Affiliations:** Unité des maladies infectieuses et tropicales (UMIT), Centre hospitalier de Cayenne

## Introduction

Les soins prodigués en zone tropicale sont complexes du fait des spécificités épidémiologiques, climatiques, culturelles et sociales. Les soignants y exerçant doivent ainsi faire preuve de capacités d'adaptation permanentes. L'objectif de ce travail était de décrire les particularités de la population soignée afin de mettre en avant en quoi le travail d'une infirmière et d'une secrétaire d'un service de consultation hospitalière de maladies infectieuses et tropicales est tout à fait singulier.

## Méthodologie

Un questionnaire a été réalisé, et proposé par l'infirmière et la secrétaire afin de recueillir des données sur le pays de naissance, la situation administrative, les langues parlées, le niveau d'alphabétisation, la profession, le type d'habitation, le mode de déplacement ainsi que le motif de la consultation. Les patients ont été inclus entre le 30 septembre 2021 et le 23 mars 2022 après le recueil d'un accord oral. Le seul critère d'exclusion était le refus de participer.

## Résultats

Cent patients ont été inclus sur une période de 6 mois. Cinquante-trois consultaient pour le suivi d'une infection virale chronique (VIH n = 38; hépatites n = 12; HTLV1 n = 3), les autres pour le suivi d'une infection aiguë. Les pays de naissance les plus représentés étaient la France (n = 34, dont la Guyane n = 26), Haïti (n = 34), le Brésil (n = 13). Trente-huit patients ne parlaient pas français (créole guyanais n = 9; anglais n = 8; portugais n = 8; créole haïtien n = 5; espagnol n = 2; autres n = 6). Le niveau scolaire était pour 29 inférieur au lycée dont 20 inférieur à l’école primaire. Parmi les patients, 49 bénéficiaient d'une prise en charge sociale précaire (CMU n = 32; AME n = 10; bon PASS n = 7), et sur les 64 nés hors de France, 11 étaient sans papiers, 14 avaient une carte de résident et 13 un titre de séjour. Trente-six patients n'avaient pas de travail ou faisaient des petits jobs et 27 vivaient en squat, location non déclarée ou étaient hébergés chez un tiers; 56 avaient des difficultés de locomotion pour se rendre à leur consultation. Enfin, 64 d'entre eux avaient recours à la pharmacopée traditionnelle.

## Discussion et conclusion

Cette étude met en exergue la grande précarité de la population prise en soins en consultation UMIT à l'hôpital de Cayenne avec un niveau socioéducatif faible. Tous ces facteurs impactent constamment la prise en soins et exigent de la part du personnel soignant et administratif, des capacités d'adaptation personnelle, d'organisation et de la disponibilité.

# Research article: Santé et recours aux soins et à la prévention des travailleuses du sexe dominicaines vivant le long du fleuve Maroni: une enquête épidémiologique transversale à visée descriptive menée en 2021

**Published:** 2023-10-31

**Authors:** Angelos GERASSIMOPOULOS, Mélanie GAILLET, Cyril ROUSSEAU, Adriana GONZALEZ, Céline MICHAUD, Nicolas VIGNIER

**Affiliations:** 1 Coordination des Centres délocalisés de prévention et de soins, Centre hospitalier de Cayenne, Guyane; 2 Croix-Rouge française, antenne de Saint-Laurent-du-Maroni, Guyane; 3 COREVIH Guyane, Centre d'investigation clinique Antilles-Guyane, CIC INSERM 1424, Centre hospitalier de Cayenne, Guyane; 4 Hôpital Avicenne, Université Sorbonne Paris Nord, IAME UMR 1137, Bobigny, Seine-Saint-Denis; 5 Institut Convergences et Migration, Aubervilliers, Seine-Saint-Denis

## Introduction

La Guyane est un territoire inégalitaire du point de vue de l'accès aux soins et à la prévention: on y observe un clivage entre le littoral et les zones de l'intérieur comme le Maroni. De nombreuses travailleuses du sexe (TDS) dominicaines exercent le long de ce fleuve transfrontalier. Une meilleure compréhension de leurs problématiques de santé ainsi qu'une connaissance approfondie de leurs conditions de vie et de travail sont susceptibles d'aider à ajuster l'offre de soins et de prévention.

## Matériels et méthodes

Une enquête épidémiologique transversale à visée descriptive a été réalisée sur une période de 12 mois auprès des 148 TDS dominicaines majeures exerçant le long du fleuve Maroni à l'aide d'un questionnaire comportant 188 questions. Nos objectifs principaux étaient de déterminer la fréquence de mésusage d'alcool, des violences subies, le taux de renoncement aux soins, la fréquence de la souffrance psychique et le recours à la prévention dans cette population.

## Résultats

Nos résultats ont mis en évidence un usage à risque d'alcool et une faible consommation d'autres drogues. L’état de santé des TDS dominicaines est globalement bon avec un recours adapté aux moyens de prévention. Des violences sont fréquemment déclarées avec peu de soutien et de moyens pour y faire face. La souffrance psychologique est fréquente et cachée, rarement prise en charge. Il y a une méconnaissance du système de santé français avec des barrières à l'origine de renoncements aux soins.

## Discussion et conclusion

Notre étude met en évidence la nécessité de sensibiliser les professionnels de santé quant aux problématiques des TDS dominicaines. Un dépistage systématique des violences, des addictions ainsi que de la souffrance psychique devrait être réalisé régulièrement. Un dispositif en lien avec les associations du territoire et l'organisation de parcours de soin dédiés sont souhaitables. Des interventions visant un « empowerment » en santé et prévention sont susceptibles d’être rapidement efficaces.

# Research article: Caractérisation des bactériémies au Centre hospitalier de Kourou en Guyane: étude pilote

**Published:** 2023-10-31

**Authors:** Arsène KPANGON, Mathilde BOUTROU, Paul LE TURNIER, Valentin KITENGE, Larissa TANGAN, Olivier PATALE, Mosa TSAFEHY, Véronique VIALETTE, Karamba SYLLA, Richard NALDJINAN, Philippe ABBOUD, Loïc EPELBOIN, Félix DJOSSOU

**Affiliations:** 1 Unité des maladies infectieuses et tropicales du Centre hospitalier Andrée Rosemon de Cayenne, Guyane; 2 Pôle médecine et urgences du Centre hospitalier de Kourou, Guyane; 3 Laboratoire Eurofins Kourou

## Introduction

Le but de notre étude était de déterminer l’écologie bactérienne des bactériémies survenues entre octobre 2021 et décembre 2022 au Centre hospitalier de Kourou.

## Méthodes

Il s'agit d'une étude pilote descriptive couvrant la période d'octobre 2021 à décembre 2022. Étaient inclus consécutivement, les patients âgés d'au moins 18 ans hospitalisés dans l'un des services du Centre hospitalier de Kourou (CHK) pour une bactériémie. La définition des bactériémies est conforme aux normes établies par le Collège des universitaires de maladies infectieuses et tropicales (CMIT) à travers la 27^e^ édition de son ouvrage de référence. Le traitement et l'analyse des données ont été réalisés manuellement. Les variables qualitatives étaient exprimées sous forme de fréquences absolues et les variables quantitatives sous la forme d'une moyenne arithmétique sans déviation standard.

## Résultats

Vingt-trois (23) isolats bactériens étaient retrouvés chez 20 patients bactériémiques. Trois (3) patients étaient décédés à l'issue de l'hospitalisation dont 2 étaient infectés par le VIH. La moyenne d’âge était de 57,5 ans. Il y avait une prédominance d'hommes soit un sex-ratio de 3. Plus de la moitié des patients venaient des zones urbaines soit 13 patients sur 20. La durée moyenne de séjour était de 23 jours. Quinze (15) isolats sur 23 appartenaient à la famille des bactéries gram-négatif. Par ordre décroissant: *Escherichia coli* était retrouvé dans 5 échantillons, *Staphylococcus* spp dans 4 échantillons et *Enterobacter cloacae* dans 3 échantillons. *Pseudomonas* spp était retrouvé dans 3 échantillons.

## Conclusion

Les bactéries les plus fréquemment rencontrées dans notre échantillon de patients bactériémiques au CHK étaient *Escherichia coli, Staphylococcus* spp, *Pseudomonas* spp, et *Enterobacter cloacae.* Une étude sur un échantillon représentatif serait utile.

# Research article: Évaluation du besoin de prise en charge kinésithérapique des patients consultant les Permanences d'accès aux soins de santé de Guyane

**Published:** 2023-10-31

**Authors:** Johanna LABURTHE, Claire BOCÉNO, Camille OBERT-MARBY, Greta CANTALUPI, Ophélie DUPART, Marwa HAJBI, Alice BELLIOT

**Affiliations:** 1 École universitaire de kinésithérapie Centre-Val de Loire (EUK-CVL), Université d'Orléans, Loiret; 2 Permanence d'accès aux soins de santé (PASS), Centre hospitalier Andrée Rosemon, Cayenne, Guyane; 3 Permanence d'accès aux soins de santé, Centre hospitalier de l'Ouest guyanais, Saint-Laurent-du-Maroni, Guyane; 4 Permanence d'accès aux soins de santé, Centre hospitalier de Kourou, Kourou, Guyane

## Introduction

La loi du 29 juillet 1998 consacre la lutte contre les exclusions comme impératif national. Cette même année sont créées les Permanences d'accès aux soins de santé (PASS), dispositifs hospitaliers permettant la prise en charge médico-sociale des personnes en situation de précarité. La Guyane est le principal département français concerné par la thématique migratoire, où 50% des Guyanais vivent sous le seuil de pauvreté. Nous connaissons déjà la fragilité de l’état de santé d'une population précaire. Nombreuses sont les pathologies y étant retrouvées nécessitant une rééducation. Rééducateurs Solidaires (RS), anciennement Kinésithérapeutes du Monde (KDM), est une association créant des réseaux entre rééducateurs bénévoles et PASS. Cependant pour cela, un état des lieux du besoin d'intervention kinésithérapique chez les patients y étant pris en charge doit être diagnostiqué. Cette étude a pour but d’étudier ce besoin dans les PASS de Guyane.

## Méthodes

Un questionnaire a été diffusé aux médecins des PASS de Cayenne, Kourou et Saint-Laurent-du-Maroni. Pour chaque patient venu en consultation médicale, ils devaient y indiquer l'existence ou non du besoin d'intervention kinésithérapique, le groupe de pathologie concerné et facultativement le diagnostic.

## Résultats

1038 réponses ont été obtenues, permettant aux échantillons de 2 des 3 PASS d’être représentatifs. L'existence du besoin d'intervention kinésithérapique a été relevée dans chacune de ces PASS chez environ 20% des patients. Les patients nécessitant une rééducation kinésithérapique présentaient principalement des pathologies musculosquelettiques et majoritairement des lombalgies.

## Discussion et conclusion

Étant donné l'instabilité administrative de ces patients, nous nous demandons s'il serait possible d'instaurer une rééducation sur plusieurs mois; si des kinésithérapeutes accepteraient d'intervenir bénévolement dans ces PASS guyanaises. Compte tenu du remaniement fréquent de l'organisation des PASS et des courtes durées de venue des kinésithérapeutes en Guyane, la création d'un réseau serait-elle pérenne?

# Research article: Facteurs de risque du syndrome alphagal, une étude cas-témoins en Guyane

**Published:** 2023-10-31

**Authors:** Jonas LE FORESTIER, Loïc EPELBOIN, François DELON, Geneviève GUILLOT, Théo BLAISE, Pierre COUPPIÉ, Félix DJOSSOU, Aude VALOIS

**Affiliations:** 1 Université des Antilles et de la Guyane, Faculté de médecine Hyacinthe Bastaraud, Pointe-à-Pitre, Guadeloupe; 2 Service des maladies infectieuses et tropicales, Centre hospitalier de Cayenne, Guyane; 3 Équipe EA 3593, Écosystèmes amazoniens et pathologie tropicale, Université de la Guyane, Cayenne, Guyane; 4 Centre d'investigation clinique, CIC INSERM 1424, Centre hospitalier de Cayenne, Guyane; 5 Université Aix-Marseille, INSERM, IRD, SESSTIM (Sciences économiques et sociales de la santé et traitement de l'information médicale), ISSPAM, Marseille, Bouchesdu-Rhône; 6 Direction interarmées du service de santé en Guyane (DIASS), Cayenne, Guyane; 7 Cabinet de pneumo-allergologie, Cayenne, Guyane; 8 Service de dermatologie et vénérologie, Centre hospitalier de Cayenne, Guyane; 9 Service de dermatologie et vénérologie, Hôpital d'instruction des armées Sainte Anne, Toulon, Var

## Introduction

Le syndrome alphagal (SAG) est une réaction allergique IgE-médiée au galactose-a-1,3-galactose (α-Gal), un disaccharide présent chez les mammifères non primates. Les patients atteints de SAG présentent des symptômes anaphylactiques retardés à la consommation de viande de mammifères ou de médicaments contenant l'a-Gal, 2 à 6 heures après le repas incriminé, compliquant le diagnostic. Le SAG se développe à l’âge adulte, des années après une consommation de viande sans symptômes.

## Objectif

Identifier les facteurs de risque de SAG en Guyane.

## Méthodes

Une étude cas-témoins a été réalisée en Guyane. Les patients majeurs suivis en allergologie pour une allergie IgE-médiée entre janvier 2021 et octobre 2022 ont été inclus. Les cas avaient un diagnostic de SAG confirmé: symptômes d'anaphylaxie suite à l'ingestion de viande de mammifère ou de médicament contenant l'a-Gal, et IgE spécifiques anti-Gal (< 0,1 kUA/mL). Les données étaient récoltées par questionnaire standardisé et les IgE spécifiques anti-Gal mesurées par tests immunoCAP.

## Résultats

Parmi 81 sujets évalués, 18 cas et 36 témoins ont été inclus. Tous les cas étaient mordus par une tique au moins une fois, contre 8 (22%) des témoins (OR = 124,1 [IC95%: 14,3-16414,9], p < 0,001). Aucun cas n’était du groupe B ou AB, contre 6 (17%) témoins (OR = 0,1 [IC95%: 0,0-1,4], p = 0,07). Un emploi en extérieur était prédictif de SAG (OR = 14,1 [IC95%: 3,9-59,3], p < 0,001) contrairement à l'atopie (OR = 0,7 [IC95%: 0,2-2,4], p = 0,59).

## Conclusion

Il était admis que la Guyane était exempte de maladie liée aux tiques. Nos résultats montrent que notre territoire n'en est pas épargné, et confirment que les morsures de tiques sont un facteur de risque majeur de SAG en Guyane. Le risque de SAG était lié au travail en extérieur (forestiers, naturalistes, militaires), et questionnerait le classement du SAG en maladie professionnelle pour ces métiers – au même titre que la maladie de Lyme.

# Research article: Les facteurs de risque de la naissance prématurée en Guyane

**Published:** 2023-10-31

**Authors:** Malika LENEUVE-DORILAS, Stéphanie BERNARD, Anne FAVRE, Alphonse LOUIS, Mathieu NACHER

**Affiliations:** 1 Service de gynécologie-obstétrique, Centre hospitalier de Cayenne, Guyane; 2 Réseau Périnat, Cayenne, Guyane; 3 Centre d'investigation clinique, INSERM 1424, Centre hospitalier de Cayenne

## Contexte et objectif

La Guyane, département-région d'outre-mer, compte près de 8000 naissances par année. Depuis 1992, la proportion de naissances prématurées y est importante aux alentours de 13,5%, soit presque le double de celle de la France (7%). Contrairement à la plupart des pays où une augmentation de la prématurité est observée, en Guyane, son taux est stable. Certes, on pourrait se satisfaire de cette non-augmentation, cependant, les décès liés à la périnatalité restent l'une des principales causes de mortalité prématurée dans ce département. Si en Guyane, le taux de prématurité n'augmente pas, il ne régresse pas non plus. Devant cette absence de régression, il semble important de comprendre les facteurs qui font qu'en Guyane, la prématurité reste si fréquente et si difficile à endiguer.

## Méthodologie

Ce travail de recherche se décline en quatre axes d'investigation: une étude rétrospective descriptive, à partir des données du RIGI (Registre d'issue de grossesses informatisé) 2013-2014 de 12 983 naissances viables du département; l’élaboration d'un score prédictif de prématurité à partir du RIGI 2013-2014, confronté aux données du RIGI 2015 de 6914 naissances viables; une étude étiologique cas-témoins de la grande prématurité, monocentrique, de février 2016 à janvier 2017 dans l'unique établissement de santé de type III de la Région; enfin, l'analyse du terme moyen à la naissance et de la morbi-mortalité à partir du RIG (Registre d'issue de grossesses) 2002-2007 de 35 648 naissances viables et du RIGI 2013-2014.

## Résultats et discussion

Sur la période d’étude, la proportion de naissances prématurées était de 13,5% (1755/12 983). La proportion de prématurité spontanée et induite était respectivement de 51,3% et 48,7% selon le RIGI 2013-2014. Plus de la moitié (57,2%) de la population d’étude bénéficiait de la sécurité sociale, néanmoins 9,3% (1211/12 983) n'avait aucune couverture sociale. L'absence de couverture sociale représentait un facteur de risque de prématurité avec un OR ajusté de 1,9 IC95% [1,6-2,3], p = 0,0001. De même, l'absence d'entretien prénatal tout comme celui de préparation à la naissance multiplieraient par deux le risque de naissance prématurée. D'autre part, le syndrome prééclamptique était la principale dysgravidie associée au risque de prématurité (OR ajusté de 6,7 [IC95% = 5,6-8,1], p = 0,001). Enfin, l'hypothèse assez répandue, suggérant qu'une partie du taux de prématurité élevée serait liée au fait que les bébés « noirs » seraient plus matures et que les mères « noires » d'ascendance afro-caribéenne accoucheraient physiologiquement plus tôt, ne ressortait pas dans nos analyses. En effet, il n'y avait pas de différence statistiquement significative de morbi-mortalité pour les nouveau-nés de mères d'origine afro-caribéenne et ceux de femmes caucasiennes.

## Conclusion

Les travaux réalisés ont retrouvé nombre de facteurs associés à la prématurité, pour certains déjà décrits par ailleurs. Bien qu’à l’échelle individuelle, il était impossible de prédire qui accoucherait prématurément, le poids des facteurs sociaux et du mauvais suivi de grossesse, suggéraient qu'une approche populationnelle pourrait être pertinente. Ainsi les femmes les plus vulnérables résidaient souvent dans des zones bien identifiées qui pourraient faire l'objet d'actions ciblées pour améliorer le suivi et dépister les complications. Cette problématique d'inégalités sociales de santé va bien au-delà de la prématurité et se retrouve pour presque toutes les pathologies, ce qui suggère qu'il y a des synergies à rechercher et que l’échelle populationnelle est sans doute stratégique. Le poids du syndrome pré-éclamptique comme facteur de risque de prématurité induite en Guyane pose question, car il semble nettement plus important qu'ailleurs pour des raisons qui restent à élucider.

# Research article: Motifs de recours des patients adressés par les CDPS aux urgences de Cayenne au cours de l'année 2019

**Published:** 2023-10-31

**Authors:** Marie-Eva MIOMANDRE, Cyril ROUSSEAU, Antoine ADENIS, Jean PUJO, Félix DJOSSOU, Alexis FREMERY

**Affiliations:** 1 Service d'accueil des urgences, Centre hospitalier de Cayenne, Cayenne, Guyane; 2 Département universitaire de médecine générale, Université des Antilles, Pointe-à-Pitre, Guadeloupe; 3 Coordination des Centres délocalisés de prévention et de soins, Centre hospitalier de Cayenne; 4 Centre d'investigation clinique Antilles-Guyane, CIC INSERM 1424, Cayenne; 5 DFR Santé, Université de Guyane, Cayenne; 6 CRB Amazonie, Centre hospitalier de Cayenne; 7 Unité des maladies infectieuses et tropicales, Centre hospitalier de Cayenne

## Introduction

La Guyane fait preuve d'une organisation sanitaire originale tentant de répondre à la situation géographique, à la précarité et à la pluriethnicité du territoire. Les Centres délocalisés de prévention et de soins (CDPS) assurent les soins primaires, de prévention mais aussi d'urgences dans des communes isolées parfois privées d'accès routier aux centres hospitaliers du littoral. Les pathologies à prendre en charge sont nombreuses, allant des soins de prévention à l'urgence vitale. La population est jeune et les accidents sont nombreux, notamment sur les sites d'orpaillage illégaux. Les complications cardiovasculaires des pathologies comme l'hypertension artérielle et le diabète sont importantes ainsi que les pathologies infectieuses inhérentes au climat et à la biodiversité de la Guyane. Le plateau technique peu fourni sur place et le turn-over important de professionnels entraînent un nombre important de transferts aux urgences de Cayenne. Les objectifs sont de décrire les caractéristiques démographiques, les motifs de consultation et la prise en charge aux urgences de Cayenne des patients adressés par les CDPS. Ainsi que d'identifier les patients qui pourraient bénéficier d'une optimisation du circuit de prise en charge au vu des données analysées.

## Méthodes

Étude observationnelle rétrospective du 1^er^ janvier au 31 décembre 2019 incluant tous les patients transférés des CDPS aux urgences de Cayenne. L'ensemble des données sociodémographiques, préhospitalières, hospitalières et l'orientation ont été recueillis.

## Résultats

Sur l'année 2019, 842 patients résidant en communes isolées ont été adressés par un CDPS aux urgences de Cayenne. Le ratio H/F est de 1,27, la moyenne d’âge est de 36 ans (± 23). La classe d’âge le plus souvent transférée est celle des 25-65 ans. Les patients avaient une couverture sociale dans 82% des cas, celle-ci était plus fréquente chez les enfants. La population provenait le plus souvent des CDPS de Maripasoula et de Saint-Georges. Les deux modes de transports prépondérants sont l'hélicoptère: utilisé pour 36% des transferts et l'ambulance dans 17% des cas essentiellement à Saint-Georges (48%). Les motifs de transfert les plus fréquents, toutes communes confondues, sont les traumatismes (28%), puis les motifs abdominaux (9%), pneumologiques (9%) et infectieux (8%). Les patients ont été hospitalisés dans 71% des cas, la réalisation d'examens complémentaires, les demandes d'avis spécialisés et la prescription d'un traitement étaient significativement plus fréquentes dans le groupe des patients hospitalisés. Parmi les patients non hospitalisés, 7% n'ont pas eu d'examens complémentaires ni d'avis spécialisé requis aux urgences. Les enfants avaient des durées de séjour aux urgences significativement plus courtes que les adultes. Les retours à domicile étaient plus fréquents chez les patients en provenance de Saint-Georges.

## Discussion et conclusion

Cette étude souligne le recours important au service des urgences de Cayenne par les CDPS. Les pathologies et leurs gravités sont variées. Notre travail relève également l'utilisation majeure des moyens aériens, notamment médicalisés, initialement prévus pour la prise en charge des urgences graves. La configuration de la Guyane rend les alternatives de transports difficiles. Notre travail identifie un certain nombre d'axes accessibles à l'optimisation de l'orientation des patients dès la prise en charge en CDPS: amélioration de l’équipement, appareillage de radiologie, intégration de la télémédecine dans la pratique quotidienne notamment pour l'aide à la décision d’évacuation sanitaire.

# Research article: Étude descriptive rétrospective des cas hospitaliers de syndrome de Guillain-Barré en Guyane entre 2006 et 2019

**Published:** 2023-10-31

**Authors:** Clémentine MONTAGNAC, Aude-Marie GRAPPERON, Mathieu NACHER, Cyril ROUSSEAU, Julie PRUDHOMME, Jean-Michel CAUVIN, Balthazar NTAB, Bertrand DE TOFFOL, Loïc EPELBOIN

**Affiliations:** 1 Service de neurologie, Centre hospitalier Andrée Rosemon, Cayenne, Guyane; 2 Centre de référence des maladies neuromusculaires et de la SLA, APHM, CHU Timone, Marseille, Bouches-du-Rhône; 3 Université de Guyane, Centre d'investigation clinique Antilles-Guyane, cIc INSERM 1424, Centre hospitalier Andrée Rosemon, Cayenne; 4 Santé publique France, Cellule Guyane; 5 Département d'information médicale, Centre hospitalier Andrée Rosemon, Cayenne; 6 Département d'information médicale, Centre hospitalier Franck Joly de l'Ouest guyanais, Saint-Laurent-du-Maroni; 7 Service de maladies infectieuses, Centre hospitalier Andrée Rosemon, Cayenne

## Introduction

Le syndrome de Guillain-Barré (SGB) est une affection neurologique souvent déclenchée par un épisode infectieux. En Guyane, malgré un contexte épidémiologique favorisant sa survenue, aucune étude n'a été réalisée. L'objectif de ce travail est de décrire les cas hospitaliers de SGB entre 2006 et 2019.

## Méthodes

Étude descriptive rétrospective des cas de SGB pris en charge à l'hôpital de Cayenne (CHC) entre le 1^er^ janvier 2006 et le 31 décembre 2019 et à l'hôpital de Saint-Laurent-du-Maroni (CHOG) entre le 1^er^ janvier 2012 et le 31 décembre 2019. Un facteur déclenchant infectieux était recherché. L'incidence a été calculée. L'incidence des cas a été mise en parallèle avec les courbes épidémiques de syndrome grippal, dengue, chikungunya et Zika.

## Résultats

Trente-cinq cas de SGB sont décrits entre 2006 et 2019. L'incidence moyenne est 1,04 cas/100 000 habitants/an (IC95% 0,69-1,38), variant entre 0 et 3,57/100 000 habitants/an. Les formes cliniques les plus fréquentes sont la forme classique sensitivomotrice (71%) et motrice pure (17%); un support ventilatoire était nécessaire pour 26% des patients. Un facteur déclenchant est trouvé dans 77% des cas: syndromes fébriles 48%, gastro-intestinaux 30%, dengue-like 22%, pseudo-grippaux 19%. Une infection récente est confirmée dans 8 cas: 3 Zika, 2 chikungunya, 1 dengue, 1 toxoplasmose amazonienne, 1 maladie de Chagas.

## Discussion et conclusion

L'incidence du SGB en Guyane est probablement sous-estimée du fait d'une offre de soins insuffisante; elle reste comparable à celles de la Caraïbe et d'Amérique latine avec une proportion de formes motrices pures plus faible. Une cause infectieuse est confirmée dans seulement huit cas avec des pathologies particulières à la Guyane. Une augmentation d'incidence est observée lors des épidémies de chikungunya et de Zika. Le bilan étiologique de SGB devrait être plus standardisé et complet. La Guyane est intéressante pour explorer le lien entre maladies tropicales et neurologie.

# Research article: Évaluation des performances diagnostiques de marqueurs fongiques dans l'histoplasmose liée au VIH sur le plateau des Guyanes

**Published:** 2023-10-31

**Authors:** Aurore MOUSSIEGT, Sigrid MAC DONALD, Marie Élisabeth BOUGNOUX, Marja VAN EER, Stephen VREDEN, Tom CHILLER, Diego CACERES, Beatriz GOMEZ, Mathieu NACHER, Olivier LORTHOLARY, Antoine ADENIS

**Affiliations:** 1 Centre d'investigation clinique Antilles-Guyane, CIC INSERM 1424, Cayenne, Guyane; 2 Fondation pour l'avancement de la recherche scientifique au Suriname (SWOS), Paramaribo, Suriname; 3 Unité de parasitologie-mycologie, Hôpital Necker-Enfants malades, APHP, Université Paris Cité, Paris, Îlede-France; 4 Mycotic Diseases Branch, Centers for Disease Control and prevention, Atlanta, Georgia, États-Unis; 5 Corporación para Investigaciones Biológicas, Medellin, Colombie; 6 Universidad del Rosario, Bogota, Colombie; 7 DFR Santé, Université de Guyane, Cayenne, Guyane; 8 Service de maladies infectieuses et tropicales, IHU Imagine, Hôpital Necker-Enfants malades, APHP, Paris; 9 CRB Amazonie, Centre hospitalier de Cayenne, Cayenne, Guyane

## Introduction

Le diagnostic de l'histoplasmose chez les personnes vivant avec le VIH (PvVIH) reste difficile malgré le développement de tests antigéniques sensibles et spécifiques pour cette maladie. Ces derniers n’étant pas disponibles commercialement dans les pays endémiques, d'autres marqueurs largement disponibles pourraient s'avérer utiles dans le bilan diagnostic initial. L'objectif de cette étude était d’évaluer et de comparer les performances du ß-(1,3)-D-glucan (BDG) et du galactomannane (GM) pour le diagnostic de l'histoplasmose associée au VIH.

## Méthodes

Nous avons réalisé une étude de performances diagnostiques des tests FungitelL et Platelia™ Aspergillus à partir d’échantillons primaires de sérum congelés au sein d'une biobanque certifiée (CRB Amazonie-DC-2021-4649). Les échantillons provenaient de PvVIH hospitalisées en Guyane et au Suriname, incluses de façon consécutive, et non exposées à un traitement antifongique dans le mois précédent (étude EDIRAPHIS-NCT01884779). Tous les patients ont donné leur consentement pour la biobanque et les études ancillaires sur les marqueurs fongiques.

## Résultats

Parmi les 121 participants inclus, on retrouvait 92 cas d'histoplasmose (34 prouvés et 58 probables) et 29 contrôles négatifs. À 150 pg/mL pour le BDG et 0,5 pour le GM, la sensibilité, la spécificité, les rapports de vraisemblance positifs et négatifs étaient respectivement: 95% [IC 95%: 85-100] *vs* 90% [77-100]; 52% [34-70] vs 83% [69-97]; 2 [1,4-3] vs 5,3 [2,4-12] et 0,1 [0,01-0,7] vs 0,12 [0,03-0,45]. Les AUC des courbes ROC étaient respectivement de 0,82 [0,68-0,91] et 0,92 [0,80-0,98].

## Discussion et conclusion

Un test négatif pour le BDG exclut l'histoplasmose dans un contexte de dépistage. Toutefois, la faible spécificité du BDG, même à seuil élevé, nécessite confirmation par un test GM positif consécutif avant d'initier le traitement antifongique. Considérant les performances équilibrées et le coût nettement inférieur du GM, ce marqueur seul devrait être privilégié dans un contexte de ressources limitées.

# Research article: Rôle de la médiation en santé dans la mise en place d'une enquête autour des cas d'hantavirus dans des quartiers informels de la périphérie de Cayenne

**Published:** 2023-10-31

**Authors:** Margot OBERLIS, Paul LE TURNIER, Frédégonde ABOUT, Luxamarre SAINT-HERVÉ, Jean MARCKENSON THERASSE, Diana PEREZ-ARANGUENA, Mathilde ZENOU, Olivier LESENS, Amadou DIALLO BALDE, Yoris DEMARS, Déborah POREZ, Camille MEUNIER, Marina PALOMAR, Karl KPOSSOU, Marion GUYOT, Adrien ORTELLI, Mathilde HANGARD, Alain LEMONNIER, Solène WIEDNER-PAPIN, Francky MUBENGA, Dominique ROUSSET, Anne LAVERGNE, Jean-Luc BAUZA, Luisiane CARVALHO, Loïc EPELBOIN, Tiphanie SUCCO

**Affiliations:** 1 Croix-Rouge française de Guyane; 2 Unité des maladies infectieuses et tropicales, Centre hospitalier de Cayenne; 3 Centre d'investigation clinique Antilles-Guyane, CIC INSERM 1424, Centre hospitalier de Cayenne; 4 Santé publique France, Cellule Guyane; 5 Agence régionale de santé Guyane; 6 CNR Hantavirus, Institut Pasteur de la Guyane; 7 Guyane Promo Santé

## Contexte

En 2022, 4 cas graves d'infection à hantavirus ont été rapportés dans des quartiers informels autour de Cayenne *versus* 7 cas entre 2007 et 2021. Santé publique France (SpF), mandatée par l'Agence régionale de santé (ARS), a sollicité la Croix-Rouge française (CRf) pour mettre en œuvre une enquête autour des cas et sensibiliser les habitants aux mesures de prévention.

## Méthodes

L'enquête a été conduite dans les trois quartiers de survenue des cas et prévoyait un prélèvement sanguin et un questionnaire sur les facteurs de risque collectés par un infirmier (IDE). L’équipe opérationnelle était constituée d’1 responsable, 2 infirmiers et 3 médiateurs en santé. Plusieurs maraudes ont été réalisées par les médiateurs pour dresser un état des lieux facilitant le déploiement de l'enquête, sensibiliser les habitants sur l'hantavirus et les informer de l'enquête. Les médiateurs ont ensuite accompagné les infirmiers pour faciliter la collecte des données, puis les médecins pour faciliter la compréhension des résultats biologiques et informer à nouveau sur les mesures de prévention. Une restitution globale des résultats à l’échelle des quartiers est en cours d'organisation.

## Résultats

Lors des maraudes, les médiateurs ont sensibilisé 343 personnes, dont 319 (93%) ont souhaité participer. De plus, 274 personnes ont été incluses soit 80% des personnes sensibilisées et 31% de participants attendus initialement. Le sex-ratio H/F était de 0,9, l’âge moyen de 42 ans et 83% étaient nés en Haïti. Enfin, 227/274 personnes prélevées (83%) sont venues récupérer leurs résultats.

## Discussion et conclusion

L'implication des médiateurs à chaque étape a probablement été un facteur de réussite dans la conception des outils, leur utilisation auprès des enquêtés et *in fine* dans l'adhésion à l'enquête autour des cas. Les actions de prévention préliminaires ont permis de sensibiliser les personnes et facilité la conscientisation du risque sanitaire. L'information préalable sur le déploiement de l'enquête aux habitants a contribué à leur adhésion. La médiation en santé et l’« aller vers » semblent indispensables à la réussite d'enquêtes épidémiologiques de terrain, en particulier dans les quartiers informels.

# Research article: Diabète et précarité dans l'Ouest guyanais: étude descriptive des patients diabétiques suivis à la Permanence d'accès aux soins de santé du Centre hospitalier de l'Ouest guyanais durant ses six premiers mois d'activité

**Published:** 2023-10-31

**Authors:** Camille OBERT-MARBY

**Affiliations:** Permanence d'accès aux soins de santé, Centre hospitalier de l'Ouest guyanais, Saint-Laurent-du-Maroni

## Introduction

La Permanence d'accès aux soins de santé (PASS) mobile du Centre hospitalier de l'Ouest guyanais (CHOG) assure depuis avril 2020 un suivi médicosocial de la population précaire de Saint-Laurent-du-Maroni. La fréquentation est exponentielle, notamment pour les malades chroniques. Les Guyanais, très exposés au diabète (7,49%, contre 4,67% en France entière) sont particulièrement précaires dans l'Ouest guyanais. Notre objectif principal est de décrire les caractéristiques médicales et indicateurs de précarité des diabétiques fréquentant la PASS mobile du CHOG pendant ses six premiers mois d'activité.

## Méthodes

Enquête rétrospective unicentrique décrivant les données médicales et indicateurs de précarité des diabétiques de la PASS du CHOG du 21 avril au 29 novembre 2020. Tous les diabétiques ayant consulté au moins une fois le médecin de la PASS ont été inclus.

## Résultats

46 diabétiques inclus, soit 8,44% des patients de la PASS: un de type 1, les autres de type 2 dont 9 méconnus. Ils étaient âgés en moyenne de 53,7 ans et 65% étaient des femmes. Leur HbA1C moyenne était de 8,9%, 1/3 des diabétiques connus étaient en rupture de traitement, et 1/3 nécessitaient une insulinothérapie. 70% étaient hypertendus, 15% obèses. La majorité était surinamaise et la moitié en situation irrégulière. 2/3 n'avaient pas de couverture sociale, 2/3 ne maîtrisaient pas bien le français. 1/3 n'avaient aucune ressource financière et 2/3 aucun accès à l'eau potable à domicile.

## Discussion et conclusion

Les diabétiques de la PASS du CHOG ont un haut risque cardiovasculaire et de multiples freins à l'accès aux soins. Les consultations délocalisées permettront d’éviter certaines ruptures thérapeutiques, et seront un des maillons manquants du parcours de soins actuel, entre médecine générale libérale et diabétologie hospitalière. Reste à trouver le maillon de l'accompagnement à domicile: infirmiers pour patients sans droits, accès à l'eau, moyens de transport pour se rendre en consultation…

# Research article: Précarité alimentaire des étudiants en Guyane en 2022: un constat post-crise sanitaire préoccupant

**Published:** 2023-10-31

**Authors:** Maëlys PROQUOT, Julie BLANC, Astrid VAN-MELLE, Antoine ADENIS, Mathieu NACHER, Célia BASURKO

**Affiliations:** Centre d'investigation clinique Antilles-Guyane, CIC INSERM 1424, Centre hospitalier de Cayenne, Cayenne, Guyane

## Objectifs

L'objectif de cette étude était d'estimer la prévalence de l'insécurité alimentaire (IA) chez les étudiants de l'Université de Guyane (UG) et de décrire les profils de ces étudiants en 2022.

## Matériel et méthode

Une enquête transversale descriptive a été menée entre le 9 avril et le 20 juin 2022 auprès des étudiants inscrits à l'UG pour l'année 2021/2022. Chaque étudiant était invité à remplir un auto-questionnaire en ligne. L'IA était explorée au moyen du score Six-Item Food Security Survey Module (FSSM) dans sa version courte. Cette échelle classait les étudiants en 3 classes selon la valeur du score: sécurité alimentaire, faible sécurité alimentaire ou très faible sécurité alimentaire. Un modèle de régression logistique a été utilisé pour étudier les principaux déterminants de cette très faible sécurité alimentaire.

## Résultats

286 étudiants ont répondu au questionnaire et 276 ont été conservés pour l'analyse. Après pondération, la prévalence de la très faible sécurité alimentaire était de 50,5% [42,3-58,6]. Les étudiants en très faible sécurité alimentaire étaient significativement plus nombreux à déclarer une alimentation déséquilibrée et une mauvaise santé mentale. Ne pas avoir assez d'argent pour couvrir ses besoins, avoir été impacté financièrement par la crise Covid-19, ne pas avoir de mutuelle étaient des déterminants de cette très faible sécurité alimentaire. Mais aussi le fait de ne pas pouvoir compter sur une personne de son entourage pour un hébergement et/ou une aide matérielle et de ne pas avoir été encouragé à faire des études à l'université.

## Conclusion

Ce constat post-crise sanitaire révèle la grande vulnérabilité des étudiants vis-à-vis de l'accès à une alimentation saine, équilibrée et adaptée à leurs besoins. Cette prévalence préoccupante nous oblige à réfléchir à des stratégies d'actions d'amélioration à court terme au bénéfice des étudiants en Guyane.

# Research article: Facteurs de risque de maladie rénale chronique chez les patients diabétiques en Guyane

**Published:** 2023-10-31

**Authors:** Christopher SACAREAU, Mathieu NACHER, André NtOUTOUM, Antoine ADENIS, Marianne HOUNNOU, Marion LIEBART, Clara SALASAR CARDOSO, Jean-Markens AURELUS, Magalie DEMAR, Olivier CASSE, Samia AMOKRANEE, Jean-François CAROD, Nezha HAFSI, Nadia SABBAH

**Affiliations:** 1 Service d'endocrinologie et maladies métaboliques, Centre hospitalier de Cayenne, Cayenne, Guyane; 2 Centre d'investigation clinique Antilles-Guyane, CIC INSERM 1424, Centre hospitalier de Cayenne; 3 Laboratoire hospitalo-universitaire de parasitologiemycologie (LHUPM), Centre hospitalier de Cayenne; 4 Équipe EA 3593, Écosystèmes amazoniens et pathologie tropicale, Université de la Guyane, Cayenne; 5 Service de médecine, Centre hospitalier de l'Ouest guyanais, Saint-Laurent-du-Maroni, Guyane; 6 Laboratoire de biologie, Centre hospitalier de l'Ouest guyanais

## Introduction

La Guyane présente un contexte social et sanitaire dégradé par rapport à la France hexagonale. La précarité concerne près de 75% de la population. La prévalence du diabète est deux fois supérieure et l'insuffisance rénale terminale 45% plus prévalente. L'objectif de notre étude est de décrire le profil des patients diabétiques atteints de maladie rénale chronique en Guyane et d'en rechercher d’éventuels facteurs de risque.

## Méthode

Réalisation d'une étude observationnelle transversale multicentrique à partir de la cohorte CoDiam, 1287 patients suivis au Centre hospitalier de Cayenne, à Saint-Laurent et dans les centres de santé délocalisés ont été inclus entre mai 2019 et juin 2021.

## Résultats

La maladie rénale chronique est présente après 12 ans en moyenne d’évolution du diabète. Par comparaison avec la France hexagonale, 41% des patients diabétiques présentaient une maladie rénale chronique, soit 12% de plus, ils étaient âgés en moyenne de 56 ans, soit 10 ans plus jeunes et ils étaient dans 48% des cas en situation d'obésité, soit 7% de plus. La précarité concernait 74% des patients, l'origine étrangère concernait 45% des patients mais ni l'une ni l'autre n’était associée à la maladie rénale chronique, contrairement aux pays où le système de santé n'est pas universel.

## Conclusion

La maladie rénale chronique touche des patients diabétiques plus jeunes et plus obèses que les autres départements français sans que des facteurs de risque spécifiques, notamment sociaux, soient mis en évidence.

# Research article: Une grande campagne binationale hors les murs pour dépister le VIH, les hépatites et la syphilis à la frontière francobrésilienne en réponse à une augmentation inquiétante du VIH

**Published:** 2023-10-31

**Authors:** Margot SEAS, Bastien BIDAUD, Clémentine ÉTIENNE, Jean-Yves CATTIN, Pedro CLAUTEAUX, Roziane BARBOSA, Margaux BARBIER, Jane BORDALO MILLER, Jonailde PINHEIRO COSTA, José GOMES, Brice DAVERTON, Sophie RHODES, Céline MICHAUD

**Affiliations:** 1 Centres délocalisés de prévention et de soins, Centre hospitalier de Cayenne, Guyane; 2 Association IDsanté, Guyane; 3 Association DPAC Fronteira, Brésil; 4 Association DAAC Guyane

## Introduction

La Guyane et le Nord du Brésil sont des régions à haute prévalence pour les VIH et les IST. La prise en charge du VIH est disponible des deux côtés de la frontière grâce au projet binational Oyapock Coopération Santé. Outre les problématiques chroniques d'accès aux soins, d’échanges économico-sexuels et d'addiction, la zone a été particulièrement impactée par la pandémie de SARS-CoV-2. En 2022, les diagnostics de VIH ont été multipliés par 3 par rapport à 2020 (n = 23 *vs* 8) avec une majorité de personnes en stade SIDA. Une campagne de dépistage binationale pour le VIH, l'hépatite B, C et la syphilis a été mise en place à la frontière.

## Méthodes

Des équipes multi-partenariales de médiateurs en santé et de soignants des rives française et brésilienne ont organisé des actions de prévention associées à une offre de dépistage rapide dans les quartiers. Un questionnaire évaluant la santé sexuelle et l'impact de la Covid a été administré aux participants. Un parcours de prise en charge transfrontalière a été établi en cas de test positif.

## Résultats

En septembre 2022, une campagne de 9 jours a permis de dépister 548 personnes de 37 ans d’âge moyen. Une personne sur 10 a déclaré avoir des relations homo- ou bisexuelles. Un tiers des personnes déclaraient n'avoir jamais fait de test. La Covid n'a pas eu d'impact sur les pratiques. Seule 1 personne a été diagnostiquée positive pour l'hépatite C et 9 pour la syphilis. Une deuxième action ciblant les quartiers précaires est prévue en avril 2023.

## Discussion et conclusion

Malgré le succès et la bonne acceptabilité de cette action en population générale, il est important de maintenir les efforts de dépistage et de développer des actions plus ciblées auprès des populations clés.

# Research article: Étude du délai d'introduction des antirétroviraux en Guyane: étude observationnelle des nouveaux patients infectés par le VIH suivis de 2015 à 2021 sur les deux principaux centres hospitaliers du département, le CHC et le CHOG

**Published:** 2023-10-31

**Authors:** Emma SCHULLER, Sébastien RABIER, Nicolas VIGNIER, Aude LUCARELLI

**Affiliations:** 1 Service de médecine et d'infectiologie du Centre hospitalier de l'Ouest guyanais (CHOG), Saint-Laurentdu-Maroni, Guyane; 2 Hôpital de jour adulte, Centre hospitalier Andrée Rosemon, Cayenne, Guyane; 3 COREVIH Guyane, Centre hospitalier Andrée Rosemon, Cayenne; 4 Service de médecine et d'infectiologie et CeGIDD (Centre gratuit d'information, de dépistage et de diagnostic), Centre hospitalier de Sallanches, Haute-Savoie

## Introduction

La pandémie de VIH a conduit l'OMS à donner des recommandations internationales uniformes. En particulier celle concernant l'instauration du traitement antirétroviral (ARV) le plus rapidement possible chez tout patient vivant avec le VIH (PvVIH), quels que soient sa charge virale et son taux de CD4. Elle a recommandé en 2015 l'instauration des ARV dans les 7 jours suivant le diagnostic. Cette étude a pour but de décrire les délais d'instauration des ARV en Guyane depuis cette nouvelle recommandation et d’évaluer les facteurs influençant ce délai.

## Méthodes

Il s'agit d'une étude descriptive rétrospective de la cohorte des nouvelles Pv-VIH, entre janvier 2015 et juin 2021, dans les deux principaux centres de prise en charge de l'infection en Guyane. Elle est complétée par une étude transversale composée d'un questionnaire soumis à un échantillon de ces PvVIH suivies sur les deux centres hospitaliers. Le critère de jugement principal était le délai d'introduction des ARV entre le diagnostic sérologique et la date de première prescription. Des analyses univariées et multivariées ont été effectuées pour rechercher des facteurs influençant le délai d'introduction des ARV.

## Résultats

980 patients ont été nouvellement pris en charge en Guyane. 920 d'entre eux ont reçu un traitement par ARV. Le délai médian d'introduction des ARV était de 40 jours. Le traitement a été initié dans les 7 jours suivant le diagnostic chez 12,26% des patients (n = 120). La première consultation spécialisée a été réalisée dans les 7 jours suivant le diagnostic chez 27,9% (n = 273).

## Discussion et conclusion

Hétérogène sur le territoire, le délai d'introduction des ARV atteint insuffisamment les objectifs de l'OMS, tout comme le délai de première consultation. Le diagnostic étant fait dans près de deux tiers des cas en ambulatoire, le parcours de soins pourrait être repensé autour du lieu, du contexte de découverte et des professionnels, autant ceux impliqués dans le diagnostic que dans l'initiation des ARV. Une étude telle que celle réalisée en région parisienne, Essai IMEA055-Fast, aurait tout son sens sur la population guyanaise pour évaluer l'impact sur ce délai d'initiation des ARV et le parcours de soins.

# Research article: Cardiopathies aiguës aux urgences: épidémiologie, évaluation prospective du suivi libéral et de l'observance thérapeutique

**Published:** 2023-10-31

**Authors:** Victor TERTRE, Yves-Kénol FRANCK, Nadia SABBAH, Jean PUJO, Mathieu NACHER, Alexis FREMERY

**Affiliations:** 1 Service d'accueil des urgences, Centre hospitalier de Cayenne, Cayenne, Guyane; 2 Département universitaire de médecine générale, Université des Antilles, Pointe-à-Pitre, Guadeloupe; 3 Service de cardiologie, Centre hospitalier de Cayenne; 4 Service d'endocrinologie, Centre hospitalier de Cayenne; 5 DFR Santé, Université de Guyane, Cayenne; 6 Centre d'investigation clinique Antilles-Guyane, CIC INSERM 1424, Cayenne; 7 CRB Amazonie, Centre hospitalier de Cayenne

## Introduction

La Guyane est un territoire particulier de par sa localisation sud-américaine et son climat tropical. Sa population est caractérisée par une importante diversité culturelle, des inégalités marquées dans les domaines socio-économique, éducatif, et dans l'accès à la santé. Dans les services d'urgences de France hexagonale, les pathologies cardiovasculaires aiguës représentent une part importante des consultations avec parfois une mise en jeu du pronostic vital. L'objectif de ce travail est la description de l’épidémiologie des cardiopathies aiguës aux urgences du Centre hospitalier de Cayenne (CHC). L'objectif secondaire est l’évaluation du suivi médical, paramédical et de l'observance thérapeutique de ces patients.

## Matériel et méthodes

Il s'agit d'une étude prospective, descriptive, réalisée dans le service d'accueil des urgences (SAU) du CHC du 1^er^ mai au 31 août 2021. Tous les patients recevant un diagnostic de cardiopathie aiguë ont été inclus. Nous avons exclu les patients dont la cardiopathie était déclenchée ou associée à une autre pathologie aiguë, ainsi que les patients refusant de participer à l’étude. Au total, 155 patients ont été analysés. Le suivi médical et paramédical était évalué sur le respect des fréquences de consultation chez le médecin traitant et le cardiologue, ainsi que la réalisation d'un suivi biologique et des ECG. L’étude de l'observance était réalisée par le questionnaire de Gired.

## Résultats

La moyenne d’âge était de 63 ± 15 ans, avec une discrète prévalence masculine (sex-ratio H/F = 1,4). La majorité des patients inclus présentaient de réels critères de gravité (74% des patients inclus possèdent une classification CIMU 3). Les facteurs de risques cardiovasculaires étaient surreprésentés par rapport à la population générale, avec notamment l'hypertension (74% des patients), le diabète (32%), la dyslipidémie (34%) et l'obésité (34%). La majorité des patients inclus (86%) présentait au moins une pathologie cardiovasculaire ou un facteur de risque cardiovasculaire connu et la pathologie présentée aux urgences était connue chez 48% d'entre eux. Les insuffisances cardiaques aiguës étaient les pathologies les plus fréquentes (41%). La réalisation d'examens complémentaires intrahospitaliers concernait 98% des patients et 65% bénéficiaient d'une hospitalisation. Le taux de mortalité aux urgences et en service était de 4,5%. Le respect complet du suivi médical et paramédical était retrouvé chez seulement 23% des patients évalués, et seuls 39% des patients décrivaient une bonne observance ou la délivrance des traitements par une infirmière à domicile. Enfin le traitement, au vu des pathologies, était adapté chez seulement 48% d'entre eux selon les recommandations françaises en vigueur.

## Conclusion

Bien que leur incidence soit inférieure à celle de France hexagonale, probablement du fait d'une population beaucoup plus jeune, on observe une variation de profil épidémiologique tant au niveau de l’âge, souvent inférieur en Guyane pour les mêmes catégories de pathologies, que dans de la prévalence des divers facteurs de risques cardiovasculaires associés. Nous avons également mis en évidence un fort niveau de précarité évalué par le niveau scolaire, la couverture maladie et la situation financière. L'analyse du régime, de l'activité sportive mais surtout du suivi médical et de l'observance fait ressortir par ailleurs, une carence dans l’éducation du patient à sa maladie, principal levier à disposition du médecin traitant. Ces pistes constitueraient un solide axe de travail pour diminuer l'augmentation de l'incidence, liée au vieillissement de la population, prévisible au cours des années à venir.

# Research article: Toxoplasmoses « amazoniennes » hospitalisées ou vues en consultation en Guyane de l'adulte immunocompétent: étude de cohorte rétrospective de 2002 à 2019

**Published:** 2023-10-31

**Authors:** Nolwenn VIDAL, Jean-François FAUCHER, Romain BLAIZOT, Audrey VALDES, Vincent POMMIER DE SANTI, Aurélien MERCIER, Raymonde TAIBI, Marie-Laure DARDÉ, Magalie DEMAR

**Affiliations:** 1 Centre hospitalier universitaire de Limoges, Haute-Vienne; 2 INSERM, U1094, Institut de neuroépidémiologie tropicale, GEIST, Limoges; 3 Service de dermatologie, Centre hospitalier de Cayenne, Guyane; 4 Centre d’épidémiologie et de santé publique des armées (CESPA), Marseille, Bouches-du-Rhône; 5 Équipe opérationnelle d'hygiène hospitalière, Centre hospitalier de Cayenne; 6 Laboratoire hospitalo-universitaire de parasitologiemycologie (LHUPM), Centre hospitalier de Cayenne

## Introduction

La toxoplasmose « amazonienne » est connue depuis les années 1990 pour ses tableaux cliniques atypiques et en particulier sa sévérité chez le patient immunocompétent. L'objectif de ce travail est de faire la synthèse des cas de toxoplasmose acquise en Guyane chez l'adulte immunocompétent.

## Méthodes

Ce travail rétrospectif (2002-2019) a inclus les patients non immunodéprimés et âgés d'au moins 18 ans. Les femmes enceintes asymptomatiques avec une séroconversion ont été exclues. Le diagnostic de toxoplasmose était retenu sur une clinique compatible associée à une PCR toxoplasmose positive ou une séroconversion objectivée ou une sérologie avec IgM positives.

## Résultats

Sur un recueil de 174 patients, le sex-ratio était de 1,76 avec une moyenne d’âge de 35,8 ans chez des patients peu comorbides. Les formes pauci-symptomatiques représentaient 42,5% (74/174) avec une atteinte ganglionnaire fébrile prédominante. L'atteinte pulmonaire associée ou non au syndrome de détresse respiratoire aiguë est significativement plus importante chez les patients qui présentent une forme sévère que ceux qui présentent des formes pauci-symptomatiques (p < 0,0001). En analyse multivariée, l'ethnie amérindienne est un facteur protecteur contre les formes graves (p = 0,017 OR = 0,011 [0-0,438]). L'analyse des souches génotypées montre une homogénéité des souches malgré leur grande diversité à l'intérieur d'un pool génétique commun. Aucun facteur pronostique en lien avec la génétique des souches n'a pu être mis en évidence.

## Discussion et conclusion

Ce travail fait ressortir la part non négligeable des formes pauci-symptomatiques dans la toxoplasmose « amazonienne » jusque-là peu décrites en dehors des atteintes oculaires isolées. La diversité génétique des souches de toxoplasmose « amazonienne » n'explique pas à elle seule l'atypie des tableaux et les facteurs de l'hôte y participent. Les Amérindiens semblent être protégés vis-à-vis des formes graves, sans pouvoir déterminer la part du degré de l'exposition particulier à leur mode de vie ou celle du patrimoine génétique en lien avec une coévolution avec les souches « amazoniennes » pour expliquer cette protection.

# Research article: Usages de la médecine traditionnelle sur le Haut-Maroni, étude mixte quantitative et qualitative

**Published:** 2023-10-31

**Authors:** Alexandre VIÉ, Pierre COUPPIÉ, Marc-Alexandre TAREAU, Théo BLAISE, Romain BLAIZOT

**Affiliations:** 1 Service de médecine, Centre hospitalier de l'Ouest guyanais, Saint-Laurent-du-Maroni, Guyane; 2 Département universitaire de médecine générale, Université des Antilles, Pointe-à-Pitre, Guadeloupe; 3 Département universitaire de dermatologie, Université des Antilles, Cayenne; Centre d'investigation clinique, Centre hospitalier Andrée Rosemon, Cayenne; 4 Centre d'investigation clinique, CIC INSERM 1424, Centre hospitalier Andrée Rosemon; 5 Service de dermatologie, Centre hospitalier Andrée Rosemon

## Introduction

L'usage de la médecine traditionnelle est peu évalué chez les patients du système de santé guyanais. Les populations autochtones du Haut-Maroni semblent avoir encore un recours fréquent au tradipraticien et aux remèdes traditionnels (RT), et ceux-ci doivent être évalués pour pouvoir renforcer leur sécurité, leur qualité et leur efficacité, dans le cadre du projet OMS pour leur intégration aux systèmes de santé nationaux.

## Méthodes

Étude descriptive transversale avec recueil de données aux dispensaires de Maripasoula, Talhuen et Antecum-Pata pendant 4 mois, par la réalisation de questionnaires quantitatifs suivis d'entretiens semi-structurés traitant des caractéristiques d'usage des RT et des habitudes de consultation chez le tradipraticien.

## Résultats

82,5% de la population avaient déjà eu recours aux RT pour 42,6% au tradipraticien. Ces pratiques étaient en recul, et de nombreux patients ne souhaitaient plus y avoir recours, avec de grandes différences en fonction des communautés étudiées. Les facteurs principaux qui semblaient influencer le recours à la médecine traditionnelle et complémentaire (MT/MC) étaient l'implantation d'un dispensaire à proximité du lieu de vie et le confort qu'il apportait, la migration récente sur le territoire et l’évangélisation des communautés.

## Discussion et conclusion

Cette étude aura permis d'apporter des données précises sur le recours majeur à la MT/MC au sein des usagers du système de santé guyanais, et permettra d'adapter les conseils prodigués par les professionnels de santé en les articulant davantage avec les pratiques locales qui seront moins stigmatisées, tout en ouvrant la possibilité à d'autres études qui cibleront les pratiques dangereuses identifiées.

# Research article: Dépistage et contribution des hépatites virales au cancer primitif du foie en Guyane entre 2009 et 2020: une étude de cohorte rétrospective

**Published:** 2023-10-31

**Authors:** Erwan VO QUANG, Vincent BÉROT, Antoine ADENIS, Magalie DEMAR, Jean-François CAROD, Mathieu NACHER, Vincent MALLET

**Affiliations:** 1 Université de Paris, Groupe hospitalo-universitaire AP-HP.Centre, Hôpital Cochin Port-Royal, DMU Cancérologie et spécialités médico-chirurgicales, Service d'hépatologie, Paris, Île-de-France; 2 Service d'infectiologie, Centre hospitalier de l'Ouest guyanais, Saint-Laurent-du-Maroni, Guyane; 3 Département d’épidémiologie et de santé publique, Centre d'investigation clinique Antilles-Guyane, CIC INSERM 1424, Centre hospitalier de Cayenne, Guyane; 4 Laboratoire, Centre hospitalier de Cayenne; 5 Laboratoire de biologie, Centre hospitalier de l'Ouest guyanais,, Saint-Laurent-du-Maroni

## Introduction

Peu de données sont disponibles actuellement sur les maladies hépatiques en Guyane, notamment sur l’épidémiologie des hépatites virales chez les patients atteints de cancer ainsi que celle des cancers primitifs du foie (CPF). Notre objectif était d’étudier l’épidémiologie des hépatites virales et du CPF en Guyane.

## Méthodes

Les recherches ont été effectuées à partir des bases de données du Programme de médicalisation des systèmes d'information (PMSI) et des bases de données biologiques des deux principaux hôpitaux de Guyane (Centre hospitalier de Cayenne et Centre hospitalier de Saint-Laurent-du-Maroni) entre septembre 2009 et juillet 2020.

## Résultats

L’échantillon comportait 3903 patients, avec un âge médian de 57 (44-69) ans et un sex-ratio de 50%. Le CPF était la 13^e^ cause de cancer, tous sexes confondus. Au moins un facteur de risque hépatique était présent chez 10,3% des patients. La prévalence des infections VHB et VHC parmi les patients avec cancer était respectivement de 6,0 et 2,6%, alors que la prévalence des infections VHB et VHC parmi les patients non atteints de cancer était de 0,7 et 0,4%. L'infection VHB, les troubles de consommation d'alcool, un FIB-4 à 3,25 étaient des facteurs de risque indépendants de CPF. La contribution des infections VHB et VHC, les troubles de consommation d'alcool, le syndrome métabolique et les autres causes non virales, non métaboliques de maladie chronique du foie au CPF étaient de 26%, 20%, 5% et 2% respectivement. 51% des patients atteints de cancer et 30% des patients atteints de cirrhose décompensée n'ont pas été dépistés pour les infections VHB et VHC.

## Discussion et conclusion

Nos résultats suggèrent que les hépatites virales restent un facteur de risque majeur du CPF et sont fréquemment négligées chez les patients atteints de cancer en Guyane, y compris en présence de complications hépatiques.

# Research article: IVG itérative: comprendre le phénomène de répétition. Enquête mixte sur l’Île de Cayenne en 2017

**Published:** 2023-10-31

**Authors:** Louise VUYLSTEKER, Stéphanie BERNARD

**Affiliations:** 1 Réseau Périnat, Guyane

## Introduction

En Guyane, les IVG de rang 3 ou plus (précédées de 2 IVG ou davantage) sont plus fréquentes que dans l'Hexagone jusqu’à représenter 29% des IVG répétées. Après le recours à une première IVG, 28 femmes sur 1 000 ont à nouveau recours à l'IVG dans l'année.

## Méthodes

Cette étude mixte a pour objectif de comprendre le phénomène de répétition des IVG. Ont été incluses dans l’étude quantitative :

Les patientes cas: toute femme consultant pour la réalisation d'une IVG au Centre hospitalier de Cayenne ou au Centre de planification et d’éducation familiale (CPEF), et ayant pratiqué au moins deux IVG antérieurement, quels que soient leur terme et la méthode utilisée.

Les patientes témoins: toute femme consultant pour la réalisation d'une première IVG ou ne présentant qu'un seul antécédent d'IVG quels que soient le terme ou la technique employée.

L'appariement des patientes s'est fait selon l’âge.

## Résultats

Les femmes qui ont recours à des IVG itératives se caractérisent par: une situation conjugale plus instable, présentent des difficultés sociales, sont plus souvent de nationalité étrangère, considèrent plus souvent que leur moyen de contraception ne convient pas. Cette étude cas-témoins a établi un lien significatif entre les demandes répétées d'IVG et des violences sexuelles subies actuellement ou dans le passé (OR 1,28 [IC95% 1,26-1,31]).

## Discussion et conclusion

Différents biais de sélection sont à relever (exclusion des mineures, perdues de vue en consultation post-IVG, barrière linguistique). Cette étude gagnerait à être approfondie et étendue à l'ensemble du territoire sur un échantillon plus diversifié d'un point de vue sociodémographique. L'IVG itérative semble s'inscrire dans une série d’évènements déstructurants qui fragilisent l'identité féminine. Une instabilité relationnelle et une sexualité peu sécurisante ont un impact sur le contrôle de la fécondité. Les femmes qui ont recours à des IVG déclaraient pour la majorité avoir subi des violences, en particulier sexuelles. Un repérage des violences chez les femmes ayant réalisé plusieurs IVG semble indispensable. La répétition de l'IVG peut être liée à une insécurité, une instabilité affective ou une ambivalence face au désir de grossesse qu'il est également nécessaire de questionner en consultation.
